# Racial and Ethnic Disparities in Occult Hypoxemia Prevalence and Clinical Outcomes Among Hospitalized Patients: A Systematic Review and Meta-analysis

**DOI:** 10.1007/s11606-024-08852-1

**Published:** 2024-07-17

**Authors:** Nicholas J. Parr, Erin H. Beech, Sarah Young, Thomas S. Valley

**Affiliations:** 1 VA Evidence Synthesis Program Coordinating Center, VA Portland Health Care System, 3710 SW US Veterans Hospital Road R&D 71, Portland, OR 97239 USA; 2 VA Center for Clinical Management Research, VA Ann Arbor Healthcare System, Ann Arbor, MI USA; 3 Division of Pulmonary and Critical Care Medicine, University of Michigan, Ann Arbor, MI USA

**Keywords:** hypoxemia, diagnostic errors, healthcare disparities

## Abstract

**Background:**

There is growing concern that pulse oximeters are routinely less accurate in hospitalized patients with darker skin pigmentation, in turn increasing risk of undetected (occult) hypoxemia and adverse clinical outcomes. The aim of this systematic review and meta-analysis was to synthesize evidence on racial and ethnic disparities in occult hypoxemia prevalence and clinical impacts of undetected hypoxemia.

**Methods:**

Ovid MEDLINE, Embase, and CINAHL databases were searched for relevant articles published through January 2024. Eligible studies must have been conducted among adults in inpatient or outpatient settings and report occult hypoxemia prevalence stratified by patient race or ethnicity, or clinical outcomes stratified by patient race or ethnicity and occult hypoxemia status. Screening for inclusion was conducted independently by two investigators. Data extraction and risk of bias assessment were conducted by one investigator then checked by a second. Outcome data were synthesized using random-effects meta-analyses.

**Results:**

Fifteen primary studies met eligibility criteria and reported occult hypoxemia prevalence in 732,505 paired oximetry measurements from 207,464 hospitalized patients. Compared with White patients, occult hypoxemia is likely more common among Black patients (pooled prevalence ratio = 1.67, 95% CI 1.47 to 1.90) and among patients identifying as Asian, Latinx, Indigenous, multiracial, or other race or ethnicity (pooled prevalence ratio = 1.39, 95% CI 1.19 to 1.64). Findings from studies reporting clinical outcomes suggest that Black patients with undetected hypoxemia may experience poorer treatment delivery outcomes than White patients with undetected hypoxemia. No evidence was found from outpatient settings.

**Discussion:**

This review and included primary studies rely on self-identified race or ethnicity, which may obscure variability in occult hypoxemia risk. Findings underscore that clinicians should be aware of the risk of occult hypoxemia in hospitalized patients with darker skin pigmentation. Moreover, oximetry data from included studies suggests that the accuracy of pulse oximeters could vary substantially from patient to patient and even within individual patients.

**Trial Registration:**

PROSPERO (CRD42023402152).

**Supplementary Information:**

The online version contains supplementary material available at 10.1007/s11606-024-08852-1.

## INTRODUCTION

Pulse oximeters are used in many clinical settings and provide a noninvasive means of monitoring oxygen levels. Pulse oximeters also allow for quick decisions at the bedside in critical situations—within ambulances, clinics, and hospitals—where patients and their clinicians depend on timely and accurate information.^1^ During the COVID-19 pandemic, pulse oximeters became essential tools for monitoring patients at home, informing clinical decision-making about oxygen supplementation, hospitalization, or intensive care.

Despite their utility, pulse oximeters may over- or underestimate a patient’s arterial oxygen saturation (SaO_2_). Pulse oximeter inaccuracy is especially concerning for patient safety when pulse oximeter readings of peripheral oxygen saturation (SpO_2_) indicate a normal blood oxygen level while a patient is actually in a hypoxemic state—a situation known as occult hypoxemia. Potential clinical impacts of occult or undetected hypoxemia include delayed or inadequate treatment, premature treatment de-escalation or discharge, and ultimately, greater morbidity and mortality.^2–4^

Inaccurate pulse oximeter readings in patients with darker skin pigmentation have been observed in clinical settings for several decades. The COVID-19 pandemic heightened concern that such inaccuracies may be widespread, routinely placing patients with darker skin pigmentation at risk of occult hypoxemia and adverse clinical outcomes. Recent studies in patients with COVID-19, for example, have shown that disparities between Black and White patients in the receipt of supplemental oxygen support and potentially life-saving treatments such as dexamethasone or remdesivir were associated with differential pulse oximeter performance.^2,5^ Other studies have found that Black patients with occult hypoxemia were more likely to experience organ failure and had higher odds of mortality compared with White patients with occult hypoxemia.^3,6^

A widely discussed retrospective study^7^ published in late 2020 analyzed nearly 50,000 paired SpO_2_–SaO_2_ measurements from adults in inpatient or intensive care settings and found that the prevalence of occult hypoxemia was over three times greater among Black patients compared with White patients (11.4% versus 3.4%). Since the publication of this analysis, additional studies have examined whether occult hypoxemia prevalence differs by patient race or ethnicity in large oximetry datasets from contemporary hospital and health system settings. Several studies have also investigated whether racial or ethnic minority patients at greater risk of occult hypoxemia, in turn, have poorer treatment and clinical outcomes than patients whose hypoxemic state was detected more quickly. The aim of this review was to synthesize this evidence to clarify the extent and magnitude of racial and ethnic disparities in the prevalence of occult hypoxemia and its clinical impacts.

## METHODS

Reporting of this systematic review and meta-analysis follows 2020 PRISMA guidance.^8^ A preregistered protocol for the review can be found on the PROSPERO registry (CRD42023402152). This protocol corresponds to a parent review^9^ requested by health system and clinical leadership of the US Department of Veterans Affairs (VA) and conducted by the VA Evidence Synthesis Program Coordinating Center. The methods and results presented here focus on occult hypoxemia prevalence and related clinical outcomes, as defined in further detail below. Given the use of published aggregate data, ethics approval or oversight of this review were not required.

### Data Sources and Searches

A research librarian searched Ovid MEDLINE, Embase, and CINAHL databases for relevant studies published through January 2024. Search strategies were reviewed by the lead author prior to searching. Complete search terms and reference counts for each database are provided in the supplemental materials.

### Study Selection

Eligible studies must have been conducted among adults in inpatient or outpatient settings and report prevalence or risk of occult hypoxemia or hypoxemia-related clinical outcomes by patient race, ethnicity, or skin pigmentation level. Studies reporting occult hypoxemia prevalence were required to define occult hypoxemia as, at minimum, arterial oxygen saturation ≤ 88% despite a paired pulse oximeter reading > 88%. Stricter criteria (e.g., pulse oximeter reading > 92%) were permitted. Pulse oximeter readings must have been taken ± 10 min of arterial oxygen saturation to be considered paired. Studies of clinical outcomes associated with occult hypoxemia were eligible only when relationships between occult hypoxemia and eligible clinical outcomes were reported by both patient race or ethnicity and occult hypoxemia status. Studies were not required to test the significance of these associations across race or ethnicity groups (e.g., by including an interaction term in analytic models). Clinical outcomes of interest were (1) timing of treatment eligibility recognition, treatment discontinuation, or discharge; (2) treatment dosage or duration; or (3) in-hospital mortality. We did not include studies that investigated whether these clinical outcomes differed by patient race or ethnicity in general, and studies that induced hypoxemia in a controlled setting were also ineligible.

Titles, abstracts, and full-text articles were independently reviewed and disagreements were resolved by consensus. References of studies excluded during full-text review (with reasons for exclusion) are listed in the supplemental materials.

### Data Extraction and Quality Assessment

Patient characteristics, study methodological details, and occult hypoxemia prevalence and clinical outcome data (when reported) were abstracted from all included studies. To facilitate synthesis, reported patient race or ethnicity was coded using one of the following terms: Asian, Black, Indigenous, Latinx, White, multiracial, or other race or ethnicity. When discussing results of individual studies, however, we used the race or ethnicity descriptors employed by the original study authors (e.g., Hispanic rather than Latinx).

Study internal validity (risk of bias) was assessed using the QUIPS tool,^10^ which is designed for studies of risk or prognostic factors. Potential biases in occult hypoxemia prevalence estimates were captured in the *prognostic factor measurement* domain of the tool. We supplemented criteria for this domain with considerations specific to prevalence assessment, including the representativeness of the sampling frame and the potential of selection biases arising from the case definition, data collection, or other aspects of the sampling methodology.^11^ Risk of bias assessment and data abstraction were first completed by one investigator then checked by another. Disagreements were resolved by consensus. Complete risk of bias ratings are provided in the supplemental materials.

### Data Synthesis and Analysis

Studies varied in whether occult hypoxemia prevalence was reported in patients or in paired observations. Each type of prevalence estimate was synthesized separately. Prevalence estimates were transformed using the standard logit transformation for analysis, and back-transformed for interpretation and reporting. To facilitate comparison of occult hypoxemia prevalence between race or ethnicity groups, we also calculated and synthesized prevalence ratios.

Prevalence estimates were synthesized using meta-analytic generalized random-effects logistic models. These models were extended to multilevel models when multiple estimates from the same study were pooled. A correlation of 0.9 was assumed for nested estimates. Conventional random-effects models were used to synthesize prevalence ratios as well as adjusted odds ratios of occult hypoxemia that were reported in some studies. All meta-analyses incorporated the Knapp-Hartung method or comparable adjustment to standard errors.^12,13^ Cluster-robust confidence intervals and degrees of freedom calculated using the Satterwaithe approximation were used in multilevel meta-analysis models.

Heterogeneity in occult hypoxemia prevalence was estimated using (restricted) maximum-likelihood estimation and is presented as 95% prediction intervals. Prediction intervals approximate the range of true effects (e.g., true occult hypoxemia prevalence) across studies, providing an estimate of the magnitude and direction of effects that are likely in future studies similar to those included in a synthesis.^14^ A prediction interval encompassing values similar to the overall estimate suggests limited heterogeneity, whereas an interval that includes estimates in the same direction as the overall estimate but that vary widely in magnitude (low to high prevalence) suggests moderate heterogeneity. If a prediction interval encompasses estimates that range widely in both magnitude and direction, then substantial heterogeneity is likely present. Prediction intervals were evaluated alongside forest plots (presented in the supplemental materials) to gauge whether estimates included in a given analysis were consistent, moderately inconsistent, or highly inconsistent. Finally, because studies of occult hypoxemia prevalence were fairly homogeneous in methodological and setting characteristics and risk of bias, we did not conduct moderation analyses based on these factors. Sample characteristics (e.g., median patient age) were not examined as potential moderators because of the risk of aggregation bias.^14^ All meta-analyses were conducted using the *metafor*^15^ package for R (R Foundation for Statistical Computing, Vienna, Austria).

After synthesizing available evidence, we rated the certainty of evidence for each outcome based on the methodology and risk of bias of available studies, the consistency and precision of results, and the directness of outcomes (whether reported outcomes are relevant to patients and providers).^16,17^ We used the following general algorithm: *high certainty* evidence consisted of multiple, large studies with consistent findings at low risk of bias, and clinically relevant outcomes; *moderate certainty* evidence consisted of multiple studies with consistent findings at low to moderate risk of bias, and clinically relevant outcomes; *low certainty* evidence consisted of a single study, or multiple small studies, with moderate to high risk of bias, inconsistent findings, and/or outcomes with limited clinical relevance; and *insufficient* evidence consisted of a single study with moderate or high risk of bias, or no available studies. Conclusions using *likely* (e.g., “Occult hypoxemia is likely more common among Black patients compared with White patients”) are based on moderate certainty evidence, while those using *may* are based on low certainty evidence. When studies were judged to be too disparate in methodological or participant characteristics—or fewer than three comparable studies were available for a given outcome—we described evidence narratively.

### Role of the Funding Source

All authors are scientific staff of the US Department of Veterans Affairs, which supported this review. The authors were fully independent and individuals with responsibility for funding decisions had no role in review design; data collection, analysis, or interpretation; or reporting of findings.

## RESULTS

**Figure 1:**
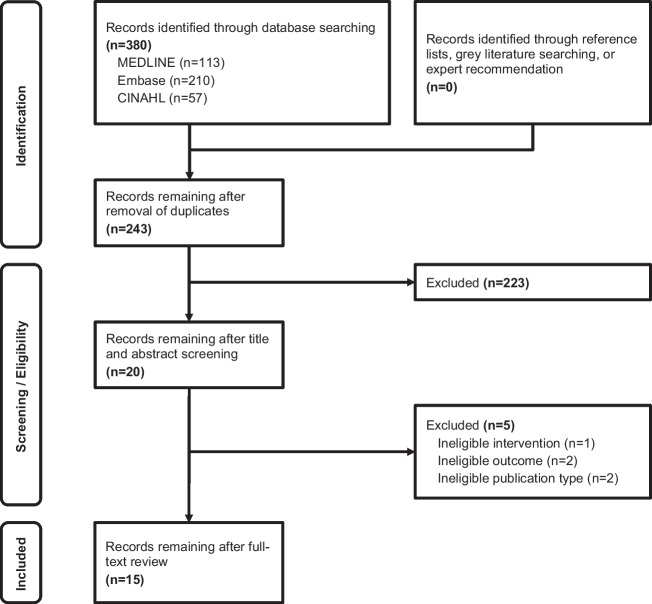
Literature flow diagram. References of studies excluded during full-text review with exclude reasons are provided in the supplemental materials. CINAHL, Cumulative Index to Nursing and Allied Health Literature.

Publications included at each stage of screening are shown in Fig. 1. Database searches resulted in 243 potentially relevant articles after deduplication, and of these, 15 studies^2,3,6,7,18–28^ met eligibility criteria. In total, studies included 732,505 paired oximetry measurements from 207,464 patients and all studies reported prevalence of occult hypoxemia stratified by self-reported race or ethnicity. Four studies^2,3,21,26^ also examined associations between occult hypoxemia and clinical outcomes by patient race or ethnicity. Characteristics of included studies are summarized in Table 1. Most newly identified studies used data from patients receiving intensive or acute care in hospitals or health systems in the USA. One study^19^ was limited to patients undergoing anesthesia, two studies^3,28^ included surgical inpatients, and two studies^2,21^ were exclusively in patients with COVID-19. No studies were conducted in an outpatient setting.

**Table 1:** Characteristics of Included Studies

Study (country) Sample size	Participants	Outcomes reported
Bangash 2022 (UK) *N* = 16,818 *N*_Obs_ = 16,818	Inpatients Median age: 63 % female: 42.1 % Black: 4.0	Prevalence of occult hypoxemia Bias and precision^﻿†^
Burnett 2022 (USA) *N* = 46,253 *N*_Obs_ = 151,070	Patients undergoing anesthesia Mean age: 57.0 % female: 45.5 % Black: 11.2	Prevalence and adjusted odds of occult hypoxemia Bias and precision
Chelsey 2022 (USA) *N* = 7693 *N*_Obs_ = 105,467	Critically ill patients admitted to ICUs Median age: 64 % female: 41.1 % Black: 25.0	Prevalence and adjusted odds of occult hypoxemia Clinical outcomes Bias and precision
Fawzy 2022 (USA) *N* = 1216 (hypoxemia) *N*_Obs_ = 32,282 *N* = 6673 (clinical outcomes)	Patients evaluated in emergency department or hospitalized for COVID-19 Mean age: 50.3–64.5 % female: 34.9–49.8 % Black: 39.3	Prevalence and adjusted odds of occult hypoxemia Clinical outcomes
Fawzy 2023 (USA) *N* = 24,504 *N*_Obs_ = 213,229	Patients hospitalized for COVID-19 Mean age: 60.9–67.5 % female: 41.9 % Black: 15.8	Prevalence and adjusted odds of occult hypoxemia Clinical outcomes
Garnet 2023 (USA) *N* = 518 *N*_Obs_ = 518	Patients with COPD undergoing oxygen testing Mean age: 69.3 % female: 3.5 % Black: 25.6	Prevalence of occult hypoxemia Bias and precision
Henry 2022 (USA) *N* = 26,603 *N*_Obs_ = 128,258	Patients admitted to ICU or undergoing surgery during inpatient hospitalization Median age: 64 % female: 41.6 % Black: 4.7	Prevalence and adjusted odds of occult hypoxemia Clinical outcomes
Kalra 2023 (USA) *N* = 196 *N*_Obs_ = 16,252	Patients on venoarterial or venovenous ECMO Median age: 47–60 % female: 37.0–44.0 % Black: 19.0–33.0	Prevalence of occult hypoxemia Bias and precision
Kalra 2023 (international registry)^*^ *N* = 13,171 *N*_Obs_ = 13,171	Patients on venovenous ECMO Median age: 49 % female: 44.0 % Black: 14.0	Prevalence of occult hypoxemia Bias and precision
Seitz 2022 (USA) *N* = 1024 *N*_Obs_ = 5557	Critically ill adults receiving mechanical ventilation (excluding patients with COVID-19) Median age: 54–58 % female: 43.0–47.0 % Black: 13.8	Prevalence of occult hypoxemia Bias and precision
Sjoding 2020 (USA) *N* = 10,001 *N*_Obs_ = 13,261	Patients receiving supplemental oxygen and patients in ICUs % Black: 13.3	Prevalence of occult hypoxemia
Sudat 2022 (USA) *N* = 13,130 *N*_Obs_ = 43,753	Hospitalized patients and patients evaluated in emergency department or hospitalized for COVID-19 Median age: 51–60 % female: 52.2–52.3 % Black: 19.5	Prevalence occult hypoxemia Bias and precision Clinical outcomes
Valbuena 2022 (USA) *N* = 28,531 *N*_Obs_ = 30,039	Inpatients (medical and surgical) Median age: 66–69 % female: 2.6–5.5 % Black: 21.7	Prevalence of occult hypoxemia Bias and precision
Valbuena 2022 ECMO (USA) *N* = 372 *N*_Obs_ = 1351	Patients in respiratory failure and about to undergo ECMO % female: 32.5 % Black: 13.7	Prevalence and adjusted odds of occult hypoxemia Bias and precision
Wong 2021 (USA) *N* = 87,971 *N*_Obs_ = 87,971	Inpatients (including ICU) Median age: 62 % female: 42.9 % Black: 29.6	Prevalence and adjusted risk of occult hypoxemia Clinical outcomes Bias and precision

*COPD* chronic obstructive pulmonary disease, *ECMO* extracorporeal membrane oxygenation, *ICU* intensive care unit,* k* number of studies, *N*_*Obs*_ number of paired observations

^*^Preprint

^†^Pulse oximeter bias and precision evidence from included studies is described in the Discussion section

**Figure 2:**
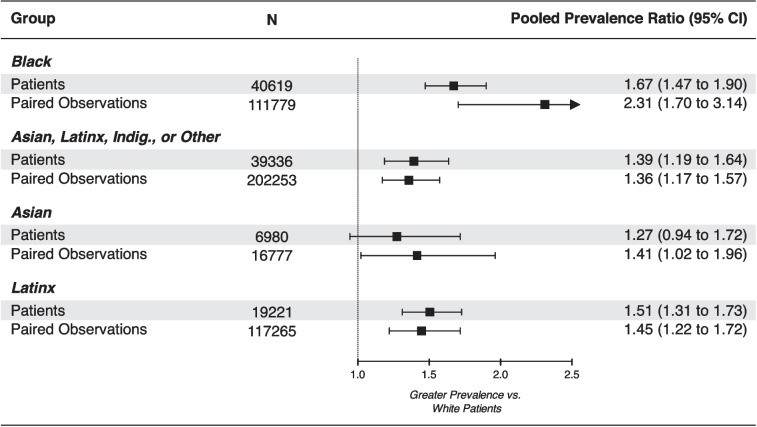
Occult hypoxemia prevalence ratios by patient race or ethnicity. Dashed line corresponds to no difference in prevalence of occult hypoxemia compared with White patients. Sample size (*N*) does not include White patients (reference group). Black patient-level prevalence ratio excludes data from one small study ^22^ for which a prevalence ratio could not be calculated (study did not report occult hypoxemia prevalence among White patients). Asian, Latinx, Indig., or Other = Asian, Latinx, Indigenous, multiracial, or other race or ethnicity.

Pooled occult hypoxemia prevalence estimates by race or ethnicity groups are presented in Table 2, and corresponding prevalence ratios are shown in Fig. 2. The prevalence of occult hypoxemia among Black patients was 9.5% (95% CI 4.7 to 18.3; *N* = 40,751), or 67% greater than the prevalence of occult hypoxemia among White patients (pooled prevalence ratio = 1.67, 95% CI 1.47 to 1.90). A larger disparity between Black and White patients was apparent at the observation level (pooled prevalence ratio = 2.31, 95% CI 1.70 to 3.14). Among patients identifying as Asian, Latinx, Indigenous, multiracial, or other race or ethnicity, the prevalence of occult hypoxemia was 8.9% (95% CI 4.0 to 18.4; *N* = 39,336), corresponding to a 39% greater prevalence of occult hypoxemia compared with White patients (pooled prevalence ratio = 1.39, 95% CI 1.19 to 1.64). Two studies^24,27^ of occult hypoxemia prevalence at the patient level did not clearly report the duration between paired SpO_2_ and SaO_2_ measurements, and one of these studies was also a preprint.^24^ Results of sensitivity analyses excluding these studies were similar to results of the full analyses (Table 2).

**Table 2:** Occult Hypoxemia Prevalence by Patient Race or Ethnicity

	*N*	Prevalence, % (95% CI)	95% prediction interval
Patients described as Black or African American			
Patients (*k* = 9)	40,751	9.5 (4.7 to 18.3)	1.0 to 52.3
Sensitivity analysis (*k* = 7)	38,923	10.0 (4.4 to 21.1)	1.0 to 55.8
Paired observations (*k* = 13)	111,779	6.0 (3.9 to 9.3)	1.1 to 26.9
Patients described as Asian, Latinx, Indigenous, multiracial, or other race or ethnicity			
Patients (*k* = 8)^*^	39,336	8.9 (4.0 to 18.4)	0.8 to 54.5
Sensitivity analysis (*k* = 6)^†^	35,579	11.1 (4.5 to 24.7)	0.9 to 62.0
Paired observations (*k* = 8)^‡^	202,253	3.4 (1.4 to 8.1)	0.2 to 34.6
Patients described as White or Caucasian			
Patients (*k* = 8)	127,377	6.4 (3.3 to 11.8)	0.9 to 33.9
Sensitivity analysis (*k* = 6)	119,419	7.3 (3.6 to 14.2)	1.1 to 35.6
Paired observations (*k* = 13)	418,473	2.5 (1.5 to 4.3)	0.3 to 16.9

*k* number of studies

^*^Eight studies reporting 19 prevalence estimates

^†^Six studies reporting 15 prevalence estimates

^‡^Eight studies reporting 20 prevalence estimates

Five studies^3,19–21,27^ of occult hypoxemia prevalence also reported odds of occult hypoxemia adjusted for potential confounders (in most cases, patient demographics, comorbidities, and treatment characteristics such as use of vasopressors) (Table 2). When pooled, Black patients had 84% greater odds of experiencing occult hypoxemia compared with White patients (pooled adjusted odds ratio = 1.84, 95% CI 1.23 to 2.75; *N* = 12,332). Reported odds ratios from all studies were similar in magnitude and consistent in direction. Four^3,19–21^ studies also included patients identifying as Asian, Latinx, Indigenous, multiracial, or other race or ethnicity, whose odds of occult hypoxemia were also significantly greater than those of White patients (pooled adjusted odds ratio = 1.30, 95% CI 1.02 to 1.66; *N* = 30,985).

All studies providing data on occult hypoxemia prevalence were rated at moderate risk of biased findings. The most common concerns were unclear detail about the pulse oximeter or CO-oximeter devices used and limitations arising from use of retrospective health record data, including unclear detail about patient characteristics and lack of control over oximetry data collection. One study^27^ that reported odds of occult hypoxemia adjusted only for patient sex and measured SpO_2_, in contrast to other studies that accounted for a more comprehensive set of potential confounders. Overall, prevalence estimates were judged to be moderately inconsistent, in that they differ in magnitude across studies but are generally consistent in direction (i.e., higher prevalences among racial or ethnic minority patient groups than among White patients). Considering both prevalence and associational findings together, evidence supporting the conclusion that occult hypoxemia is likely more common among Black patients than among White patients was rated moderate certainty. Evidence supporting the conclusion that patients identifying as Asian, Latinx, Indigenous, multiracial, or other race or ethnicity likely experience occult hypoxemia more frequently than White patients (but not as frequently as Black patients) was also considered moderate certainty.

Four observational studies^2,3,6,21^ were found that examined whether disparities in occult hypoxemia were associated with elevated risk of adverse clinical or healthcare utilization outcomes. An additional study,^26^ not described below, reported a causal inference analysis of clinical impacts of differential pulse oximeter measurement error (rather than occult hypoxemia per se) between Black and White patients.

At present, the largest available study of racial and ethnic disparities in clinical outcomes of occult hypoxemia is a US study^6^ that examined health record data from Black, Hispanic, Asian, and White acute care patients (*N* = 87,971) seen in 215 hospitals and 382 intensive care units. The prevalence of occult hypoxemia (defined as SaO_2_ < 88% despite a matched SpO_2_ > 88%) significantly differed across race or ethnicity groups (*p* < 0.001) and was highest among Black patients (6.9%; *N* = 26,032). After adjusting for patient age, sex, and Sequential Organ Failure Assessment (SOFA) score, Black patients with occult hypoxemia experienced significantly shorter average length of stay compared with Black patients without occult hypoxemia (− 3.0 days, *p* < 0.01). Length of stay for White patients (*N* = 57,623) with occult hypoxemia was also significantly shorter than for White patients without occult hypoxemia, but by only 0.5 days on average (*p* < 0.01). Length of stay among Asian (*N* = 1919) and Hispanic (*N* = 2397) patients with and without occult hypoxemia also differed by less than 1 day on average. In-hospital mortality was more common in patients experiencing occult hypoxemia regardless of race or ethnicity, with the largest difference among White patients (11.1% greater than White patients without occult hypoxemia, *p* < 0.001). The study did not test whether associations between occult hypoxemia and clinical outcomes significantly differed across race or ethnicity groups.

A second study^3^ used health record data from 26,603 patients in intensive care and surgery settings at three US academic medical centers, finding that Black patients had 65% greater odds of experiencing occult hypoxemia than White patients after adjusting for patient sex, treatment characteristics, comorbidities, and SpO_2_ (adjusted odds ratio = 1.65, 95% CI 1.28 to 2.14). In turn, patients with occult hypoxemia experienced significantly greater in-hospital mortality in both surgical settings (adjusted odds ratio = 2.96, 95% CI 1.20 to 7.28) and intensive care units (adjusted odds ratio = 1.36, 95% CI 1.03 to 1.80). Among surgery patients, occult hypoxemia was also significantly associated with fewer hospital-free days (− 2.5 days, 95% CI − 3.9 to − 1.2). Analyses of in-hospital mortality and hospital-free days were adjusted for patient age, sex, comorbidities, acuity, and setting. An interaction of occult hypoxemia status and patient race or ethnicity was nonsignificant (*p*-value not reported). Black, Asian, or American Indian patients together made up a relatively small proportion of the patient sample (2110 versus 24,493 White patients).

Another study^21^ examined clinical outcomes of unrecognized treatment need (defined as an admission SaO_2_ < 94% despite a matched SpO_2_ > 94%) among patients hospitalized for COVID-19 at 186 US acute care facilities (*N* = 8,635 in clinical outcomes analysis). After adjusting for demographic characteristics and potential clinical confounders, Black and Hispanic patients had significantly greater odds of unrecognized need for COVID-19 therapy compared with White patients (Black patients: adjusted odds ratio = 1.46, 95% CI 1.23 to 1.72; Hispanic patients: adjusted odds ratio = 1.18, 95% CI 1.01 to 1.39). Patients with unrecognized treatment need were somewhat less likely to receive COVID-19 therapy than patients with initially recognized treatment need (adjusted hazard ratio = 0.90, 95% CI 0.83 to 0.97) and had significantly greater odds of 30-day hospital readmission (adjusted odds ratio = 2.41, 95% CI 1.39 to 4.18). Average length of stay appeared to be shorter for patients with unrecognized treatment need compared with patients with recognized treatment need (− 1.4 days, 95% CI, − 3.1 to 0.2), but this difference was nonsignificant. In-hospital mortality was similar regardless of treatment recognition (adjusted odds ratio = 0.84, 95% CI 0.71 to 1.01). As in the previous study, an interaction with patient race or ethnicity was nonsignificant (*p*-values = 0.45 and 0.14, respectively), though median time to receipt of therapy was 2.1 to 4.5 h longer among Black patients with unrecognized treatment need compared with patients of any other race or ethnicity (regardless of whether they had unrecognized or recognized treatment need). Unrecognized treatment need was not significantly associated with in-hospital mortality or length of stay in this study.

Finally, a comparatively small study^2^ in patients evaluated in the emergency department or hospitalized for COVID-19 in a US health system (*N* = 1903 in clinical outcomes analysis) examined whether patients predicted to have an SaO_2_ of 94% or less prior to a measured SpO_2_ of 94% or less (i.e., an unrecognized hypoxemic state) experienced delayed recognition of treatment eligibility or delayed treatment initiation. Occult hypoxemia (defined as SaO_2_ < 88% despite a concurrent SpO_2_ between 92 and 96%) was most prevalent among Black, Hispanic, and Asian patients (28.5–30.2%) compared with White patients (17.2%) (*p-*values not reported). Failure to recognize eligibility or delayed recognition of eligibility was significantly more likely among both Black patients (adjusted hazard ratio = 0.71, 95% CI 0.63 to 0.80) and Hispanic patients (adjusted hazard ratio = 0.77, 95% CI 0.66 to 0.89) with unrecognized hypoxemia compared with White patients with unrecognized hypoxemia. Analyses were adjusted for demographics, comorbidities, acuity, and laboratory values (e.g., hemoglobin). In the subset of patients eventually recognized as treatment eligible, the median delay to eligibility recognition was about 2 h longer for Black patients and Asian patients compared with Hispanic and White patients (*p* = 0.01 for Black versus White patients).

Available evidence was considered insufficient to make firm conclusions about disparities in clinical impacts of occult hypoxemia, given that few studies were available and those that were identified differed in outcome definitions, analytic approaches, and setting and sample characteristics. Additionally, studies generally assessed clinical impacts of occult hypoxemia indirectly, by first examining occult hypoxemia prevalences then modeling associations between occult hypoxemia status and clinical outcomes separately. Nonetheless, these studies are fairly consistent in showing that patients with occult hypoxemia have worse outcomes than patients without occult hypoxemia, and they provide suggestive evidence that Black patients with undetected hypoxemia may experience poorer treatment delivery outcomes than White patients with undetected hypoxemia.

## DISCUSSION

Occult hypoxemia is likely more common among Black patients compared with White patients. Patients identifying as Asian, Latinx, Indigenous, multiracial, or other race or ethnicity also likely experience occult hypoxemia more frequently than White patients, but less frequently than Black patients. Studies examining differences in clinical outcomes associated with occult hypoxemia are methodologically inconsistent but provide suggestive evidence that Black patients with undetected hypoxemia could experience poorer treatment delivery outcomes than White patients with undetected hypoxemia. Available evidence on disparities in occult hypoxemia prevalence and outcomes is based on oximetry data from pulse oximeters currently in wide use, so it is conceivable that differential pulse oximeter inaccuracy due to patient skin pigmentation level is commonplace in modern healthcare delivery. At the same time, even larger disparities in occult hypoxemia might be expected if pulse oximeters overestimate oxygen saturation among racial and ethnic minority patients by a *consistently* large magnitude. This suggests the presence of considerable variability in the amount of bias in pulse oximeter readings between (and potentially within) individual patients.

A recent systematic review and meta-analysis^29^ synthesized pulse oximeter accuracy data from 1990 to the early COVID-19 pandemic and found that the average amount of bias in pulse oximeter readings was somewhat higher for Black patients than for White patients (1.52 versus 0.55 from a total of 18,623 paired observations; see Table 3). Precision, or the variability in the amount of bias across patients, was similar between groups and ranged from 1.55 to 1.68. To compare these findings with the much larger amount of pulse oximeter accuracy data generated in contemporary hospital settings during and after the COVID-19 pandemic, we pooled mean bias and precision data from the 11 included studies that reported these data.^6,18–20,22–28^ These studies contribute 455,867 new paired oximetry observations from 214,715 patients. Details of our analytic approach to pooling accuracy data are provided in the supplemental materials.

**Table 3:** Pooled Estimates of Pulse Oximeter Mean Bias and Precision by Patient Race or Ethnicity

	*N*	*N*_Obs_	Mean bias (95% CI)	Precision (95% CI)
Patients described as Black or African American				
Recent evidence^*^	44,757	90,399	1.16 (0.27 to 2.06)	4.48 (3.10 to 6.49)
Earlier evidence^†^	459	5753	1.52 (0.95 to 2.09)	1.68 (1.32 to 2.14)
Patients described as Asian, Latinx, Indigenous, Multiracial, or other race or ethnicity				
Recent evidence^*^	31,339	84,691	0.85 (0.04 to 1.66)	3.84 (2.53 to 5.83)
Earlier evidence^†^	522	2646	0.31 (0.09 to 0.54)	1.55 (0.53 to 4.53)
Patients described as White or Caucasian				
Recent evidence^*^	138,619	280,777	0.44 (-0.22 to 1.09)	3.80 (2.80 to 5.17)
Earlier evidence^†^	2195	12,870	0.55 (-0.21 to 1.31)	1.55 (1.31 to 1.82)

*N*_*Obs*_ number of paired observations

^*^Derived from pulse oximeter accuracy data reported in 11 included studies published between November 2021 and November 2023

^†^Pooled estimates calculated by Shi et al,^29^ ﻿based on 14 studies of oximetry data collected from 1990 through late 2020. One small study [27] (*N* = 372) is included in both earlier and recent evidence.

As shown in Table 3, although modern pulse oximeters appear to overestimate blood oxygen saturation in Black patients compared with White patients, the magnitude of bias is on average fairly small across race or ethnicity groups. This observation is consistent with findings from the earlier review of pre-COVID-19 evidence.^29^ However, in contrast with older studies that generally found modest variability is the amount of bias across patients (precision), evidence from more recent and much larger studies indicates that the accuracy of pulse oximeter readings could vary substantially from patient to patient regardless of their race or ethnicity. Moreover, findings from a recent analysis^28^ of 30,000 paired oximetry measurements from the US Veterans Health Administration suggest there may also be considerable variability in pulse oximeter bias *within* individual patients. Investigators found that even when Black and White patients were not experiencing occult hypoxemia at their first oximetry reading of a given day, Black patients were more likely to be experiencing occult hypoxemia at a second oximetry reading on the *same day* (i.e., Black patients’ probability of occult hypoxemia was more variable among same-day oximetry readings than that of White patients).

When pulse oximeters provide faulty data to clinicians, patients are at increased risk of harm—particularly when measurements are falsely reassuring as they are with occult hypoxemia. At present, the US Food and Drug Administration (FDA) recommends pre-market studies used for approval of new pulse oximeter devices include only two darkly pigmented patients (or 15% of the participant sample, whichever is larger).^30,31^ Moreover, as stated by the FDA, “these clinical studies are neither recommended to enroll demographic groups nor be statistically powered to detect differences in performance between such cohorts”.^31^ In response to ongoing concerns about disparities in pulse oximeter accuracy and regulatory requirements for pulse oximeter approval, the FDA has convened two public meetings of its Anesthesiology and Respiratory Therapy Devices Panel since late 2022.^31–33^ The Panel released a discussion paper in November 2023 that proposes several steps to improve pre-market pulse oximeter studies.^31^ These include enrollment of a larger number of participants; ensuring that a minimum number of participants are enrolled across all values of a validated skin tone scale, corroborated by an objective skin pigmentation measurement at the pulse oximeter sensor site; and use of a more sophisticated analytic approach to evaluate oximeter performance data.^31^ Other recent recommendations have included testing oximeters under real-world healthcare conditions and incorporating perfusion into validation requirements.^20,34^

In the nearer term, reassessing oxygen saturation in arterial blood more routinely—particularly for patients who show signs or symptoms of arterial hypoxemia—has been proposed as one step to reduce the risk of undetected hypoxemia.^28^ Another approach suggested to mitigate this risk is to raise the target oxygen saturation range for all patients from 92–96% to 94–98%, though this may increase risk of hyperoxemia and use of supplemental oxygen among patients who may not require it.^20^ Applying a fixed skin tone-based correction factor to readings from currently available pulse oximeters has also been discussed, but such adjustments have a controversial history^35–37^ and may have limited efficacy because the accuracy and reliability of pulse oximetry readings are influenced by multiple factors that cannot be accounted for in a single correction factor.^36,38^

Although changes to clinical practice may help to offset the impact of pulse oximeter inaccuracies on hypoxemia detection, it is clear that significant advancements in noninvasive oximeter technology are needed. Improving pulse oximeter technology is an active research area. A recent validation study,^39^ for example, tested an investigational noninvasive oximeter that uses green rather than the conventional red light, targets superficial skin layers to increase sensitivity to tissue hypoxia, and implements patient-specific skin tone calibration (rather than a fixed correction factor). The study enrolled equal proportions of patients with fair, brown, and dark skin tones based on the Von Luschan Chromatic Scale. Oxygen saturation readings from the novel oximeter were more highly correlated with blood-based oximetry (*r* = 0.76) than pulse oximeter readings (*r* = 0.47), and the device was also able to accurately assess oxygen levels in cases in which the pulse oximeter failed, including a patient with very dark skin tone.

As noted above, recent studies that contribute most available occult hypoxemia data report their results by patient-identified race or ethnicity (likely due to the use of patient health record data). A concern with the use of self-identified race or ethnicity—versus objective skin pigmentation level—is that it may introduce spurious variation across studies and lead to unexpected or clinically counterintuitive findings, given that individuals with a wide range of skin pigmentation levels could identify with the same race or ethnicity. The present review shares this limitation, in that categorizing individuals according to self-identified race or ethnicity may obscure variation in occult hypoxemia risk. This limitation is potentially exacerbated by aggregating results for patients identifying as Asian, Latinx, Indigenous, multiracial, or other race or ethnicity for syntheses, which was necessary because of the more limited representation of individuals with these races or ethnicities in available data. Additionally, limits to the extent of oximetry data captured in electronic health records meant that it was not feasible for us to examine whether pulse oximeter device type impacted occult hypoxemia risk, and it is also possible that time-stamps used to define paired observations may be inaccurately recorded in some cases. Despite these limitations, findings of this review underscore that clinicians should be aware of the risk of occult hypoxemia in patients with darker skin pigmentation and the potential of clinically important variability in pulse oximeter accuracy across and within patients.

## Supplementary Information

Below is the link to the electronic supplementary material.

Supplementary materials (DOCX 1744 KB)

## Data Availability

Analytic data are available by request to the corresponding author.

## References

[1] **Luks AM, Swenson ER**. Pulse oximetry for monitoring patients with COVID-19 at home: potential pitfalls and practical guidance. Ann Am Thorac Soc. 2020;17(9): 1040–1046. 10.1513/AnnalsATS.202005-418FR. 10.1513/AnnalsATS.202005-418FR PMC7462317 32521167

[2] **Fawzy A, Wu TD, Wang K, Robinson ML, Farha J, Bradke A, et al**. Racial and ethnic discrepancy in pulse oximetry and delayed identification of treatment eligibility among patients with COVID-19. JAMA Intern Med. 2022;182(7): 730–738. 10.1001/jamainternmed.2022.1906. 10.1001/jamainternmed.2022.1906 PMC9257583 35639368

[3] **Henry NR, Hanson AC, Schulte PJ, Warner NS, Manento MN, Weister TJ, et al**. Disparities in hypoxemia detection by pulse oximetry across self-identified racial groups and associations with clinical outcomes. Crit Care Med. 2022;50(2): 204–211. 10.1097/CCM.0000000000005394. 10.1097/CCM.0000000000005394 PMC9070439 35100193

[4] **Gadrey SM, Mohanty P, Haughey SP, Jacobsen BA, Dubester KJ, Webb KM, et al**. Overt and occult hypoxemia in patients hospitalized with COVID-19. Crit Care Exp. 2023;5(1): e0825. 10.1097/CCE.0000000000000825. 10.1097/CCE.0000000000000825 PMC9857543 36699241

[5] **Gottlieb ER, Ziegler J, Morley K, Rush B, Celi LA**. Assessment of racial and ethnic differences in oxygen supplementation among patients in the intensive care unit. JAMA Intern Med. 2022;182(8): 849. 10.1001/jamainternmed.2022.2587. 10.1001/jamainternmed.2022.2587 PMC9274443 35816344

[6] **Wong AKI, Charpignon M, Kim H, Josef C, de Hond AAH, Fojas JJ, et al**. Analysis of discrepancies between pulse oximetry and arterial oxygen saturation measurements by race and ethnicity and association with organ dysfunction and mortality. JAMA Netw Open. 2021;4(11): e2131674. 10.1001/jamanetworkopen.2021.31674. 10.1001/jamanetworkopen.2021.31674 PMC9178439 34730820

[7] **Sjoding MW, Dickson RP, Iwashyna TJ, Gay SE, Valley TS**. Racial bias in pulse oximetry measurement. N Engl J Med. 2020;383(25): 2477–2478. 10.1056/NEJMc2029240 10.1056/NEJMc2029240 PMC7808260 33326721

[8] **Page MJ, McKenzie JE, Bossuyt PM, Boutron I, Hoffmann TC, Mulrow CD, et al**. The PRISMA 2020 statement: an updated guideline for reporting systematic reviews. J Clin Epidemiol. 2021;134: 178–189. 10.1016/j.jclinepi.2021.03.001. 10.1016/j.jclinepi.2021.03.001 33789819

[9] **Parr NJ, Beech EH, Young S**. Differential pulse oximeter accuracy, occult hypoxemia prevalence, and clinical outcomes by patient race/ethnicity: a systematic review. VA ESP Project #09–199, 2023. https://www.hsrd.research.va.gov/publications/esp/pulse-oximetry.cfm 38598648

[10] **Hayden JA, van der Windt DA, Cartwright JL, Pierre Côté DC, Bombardier C**. Assessing bias in studies of prognostic factors. Ann Intern Med. 2013;158(4): 280–286. 10.7326/0003-4819-158-4-201302190-00009. 10.7326/0003-4819-158-4-201302190-00009 23420236

[11] **Hoy D, Brooks P, Woolf A, Blyth F, March L, Bain C, et al**. Assessing risk of bias in prevalence studies: modification of an existing tool and evidence of interrater agreement. J Clin Epidemiol. 2012;65(9): 934–939. 10.1016/j.jclinepi.2011.11.014. 10.1016/j.jclinepi.2011.11.014 22742910

[12] **Knapp G, Hartung J**. Improved tests for a random effects meta-regression with a single covariate. Stat Med. 2003;22(17): 2693–2710. 10.1002/sim.1482. 10.1002/sim.1482 12939780

[13] **Rover C, Knapp G, Friede T**. Hartung-Knapp-Sidik-Jonkman approach and its modification for random-effects meta-analysis with few studies. BMC Med Res Methodol. 2015;15: 99. 10.1186/s12874-015-0091-1. 10.1186/s12874-015-0091-1 PMC4647507 26573817

[14] **Parr NJ, Schweer-Collins ML, Darlington TM, Tanner-Smith EE**. Meta-analytic approaches for examining complexity and heterogeneity in studies of adolescent development. J Adolesce. 2019;77:168–178. 10.1016/j.adolescence.2019.10.009 10.1016/j.adolescence.2019.10.009 PMC6934259 31739275

[15] **Viechtbauer W**. metafor: Meta-analysis package for R. The Comprehensive R Archive Network; 2023.

[16] **Guyatt G, Oxman AD, Akl EA, Kunz R, Vist G, Brozek J, et al**. GRADE guidelines: 1. introduction-GRADE evidence profiles and summary of findings tables. J Clin Epidemiol. 2011;64(4): 383–394. 10.1016/j.jclinepi.2010.04.026. 10.1016/j.jclinepi.2010.04.026 21195583

[17] **Berkman ND, Lohr KN, Ansari M, McDonagh M, Balk E, Whitlock E, et al**. Grading the strength of a body of evidence when assessing health care interventions for the Effective Health Care Program of the Agency for Healthcare Research and Quality: an update. In: Methods Guide for Effectiveness and Comparative Effectiveness Reviews. Agency for Healthcare Research and Quality, Rockville, MD. 2013. https://www.ncbi.nlm.nih.gov/books/NBK174881/24404627

[18] **Bangash MN, Hodson J, Evison F, Patel JM, Johnston AM, Gallier S, et al**. Impact of ethnicity on the accuracy of measurements of oxygen saturations: a retrospective observational cohort study. EClinicalMedicine. 2022;48. 10.1016/j.eclinm.2022.101428. 10.1016/j.eclinm.2022.101428 PMC9096912 35706489

[19] **Burnett GW, Stannard B, Wax DB, Lin HM, Pyram-Vincent C, DeMaria S, et al**. Self-reported race/ethnicity and intraoperative occult hypoxemia: a retrospective cohort study. Anesthesiology. 2022;136(5): 688–696. 10.1097/ALN.0000000000004153. 10.1097/ALN.0000000000004153 35231085

[20] **Chesley CF, Lane-Fall MB, Panchanadam V, Harhay MO, Wani AA, Mikkelsen ME, et al**. Racial disparities in occult hypoxemia and clinically based mitigation strategies to apply in advance of technological advancements. Respir Care. 2022;67(12): 1499–1507. 10.4187/respcare.09769. 10.4187/respcare.09769 35679133

[21] **Fawzy A, Wu TD, Wang K, Sands KE, Fisher AM, Arnold Egloff SA, et al**. Clinical outcomes associated with overestimation of oxygen saturation by pulse oximetry in patients hospitalized with COVID-19. JAMA Netw Open. 2023;6(8): e2330856. 10.1001/jamanetworkopen.2023.30856. 10.1001/jamanetworkopen.2023.30856 PMC10450566 37615985

[22] **Garnet B, Diaz-Lankenau R, Jean E, Campos M**. Accuracy of pulse oximetry for long-term oxygen therapy assessment in chronic obstructive pulmonary disease. Ann Am Thorac Soc. 2023;20(11): 1587–1594. 10.1513/AnnalsATS.202209-837OC. 10.1513/AnnalsATS.202209-837OC 37413976

[23] **Kalra A, Shou BL, Zhao D, Wilcox C, Keller SP, Whitman GJR, et al**. Racial and ethnical discrepancy in hypoxemia detection in patients on extracorporeal membrane oxygenation. JTCVS Open. 2023;14: 145–170. 10.1016/j.xjon.2023.02.011. 10.1016/j.xjon.2023.02.011 PMC10328809 37425474

[24] **Kalra A, Wilcox C, Holmes SD, Tonna JE, Jeong IS, Rycus P, et al**. Characterizing the racial discrepancy in hypoxemia detection in VV-ECMO: an ELSO registry analysis. 2023. 10.21203/rs.3.rs-3617237/v1. 10.1007/s00408-024-00711-4 PMC11456976 38856932

[25] **Seitz KP, Wang L, Casey JD, Markus SA, Jackson KE, Qian ET, et al**. Pulse oximetry and race in critically ill adults. Crit Care Explor. 2022;4(9): e0758. 10.1097/CCE.0000000000000758 10.1097/CCE.0000000000000758 PMC9478292 36128001

[26] **Sudat SEK, Wesson P, Rhoads KF, Brown S, Aboelata N, Pressman AR, et al**. Racial disparities in pulse oximeter device inaccuracy and estimated clinical impact on COVID-19 treatment course. Am J Epidemiol. 2023;192(5): 703–713. 10.1093/aje/kwac164 10.1093/aje/kwac164 PMC9619495 36173743

[27] **Valbuena VSM, Barbaro RP, Claar D, Valley TS, Dickson RP, Gay SE, et al**. Racial bias in pulse oximetry measurement among patients about to undergo extracorporeal membrane oxygenation in 2019-2020: a retrospective cohort study. Chest. 2022;161(4): 971–978. 10.1016/j.chest.2021.09.025. 10.1016/j.chest.2021.09.025 PMC9005857 34592317

[28] **Valbuena VSM, Seelye S, Sjoding MW, Valley TS, Dickson RP, Gay SE, et al**. Racial bias and reproducibility in pulse oximetry among medical and surgical inpatients in general care in the Veterans Health Administration 2013-19: multicenter, retrospective cohort study. BMJ. 2022;378: e069775. 10.1136/bmj-2021-069775. 10.1136/bmj-2021-069775 PMC9254870 35793817

[29] **Shi C, Goodall M, Dumville J, Hill J, Norman G, Hamer O, et al**. The accuracy of pulse oximetry in measuring oxygen saturation by levels of skin pigmentation: a systematic review and meta-analysis. BMC Med. 2022;20(1): 267. 10.1186/s12916-022-02452-8. 10.1186/s12916-022-02452-8 PMC9377806 35971142

[30] US Food and Drug Administration. Pulse oximeters − premarket notification submissions: guidance for industry and Food and Drug Administration staff. 2013. https://www.fda.gov/media/72470/download

[31] US Food and Drug Administration. Approach for improving the performance evaluation of pulse oximeter devices taking into consideration skin pigmentation, race and ethnicity: discussion paper and request for feedback. 2023. https://www.fda.gov/media/173905/download

[32] Anesthesiology and respiratory therapy devices panel of the medical devices advisory committee meeting announcement (November 1, 2022). 2022. https://www.fda.gov/advisory-committees/advisory-committee-calendar/november-1-2022-anesthesiology-and-respiratory-therapy-devices-panel-medical-devices-advisory#event-information

[33] Anesthesiology and respiratory therapy devices panel of the medical devices advisory committee meeting announcement (February 2, 2024). 2024. https://www.fda.gov/advisory-committees/advisory-committee-calendar/february-2-2024-anesthesiology-and-respiratory-therapy-devices-panel-medical-devices-advisory

[34] **Okunlola OE, Lipnick MS, Batchelder PB, Bernstein M, Feiner JR, Bickler PE**. Pulse oximeter performance, racial inequity, and the work ahead. Respir Care. 2022;67(2): 252–257. 10.4187/respcare.09795. 10.4187/respcare.09795 34772785

[35] **Braun L**. Race correction and spirometry: why history matters. Chest. 2021;159(4):1670–1675. 10.1016/j.chest.2020.10.046. 10.1016/j.chest.2020.10.046 33263290

[36] **Fawzy A, Valbuena VSM, Chesley CF, Wu TD, Iwashyna TJ**. Dynamic errors in pulse oximetry preclude use of correction factor. Ann Am Thorac Soc. 2022;20(2):338–339. 10.1513/AnnalsATS.202210-872LE. 10.1513/AnnalsATS.202210-872LE PMC9989867 36442148

[37] **Vyas DA, Eisenstein LG, Jones DS**. Hidden in plain sight — reconsidering the use of race correction in clinical algorithms. N Engl J Med. 2020;383(9): 874–882. 10.1056/NEJMms2004740. 10.1056/NEJMms2004740 32853499

[38] **Jamali H, Castillo LT, Morgan CC, Coult J, Muhammad JL, Osobamiro OO, et al**. Racial disparity in oxygen saturation measurements by pulse oximetry: evidence and implications. Ann Am Thorac Soc. 2022;19(12):1951–1964. 10.1513/AnnalsATS.202203-270CME. 10.1513/AnnalsATS.202203-270CME 36166259

[39] **Gokhale SG, Daggubati V, Alexandrakis G**. Innovative technology to eliminate the racial bias in non-invasive, point-of-care (POC) haemoglobin and pulse oximetry measurements. BMJ Innov. 2023;9(2):73–77. 10.1136/bmjinnov-2022-001018.

