# Supplementary material for: Racial and Ethnic Disparities in Occult Hypoxemia Prevalence and Clinical Outcomes Among Hospitalized Patients: A Systematic Review and Meta-analysis
Source: J Gen Intern Med. 2024 Jul 17;39(13):2543–53. doi: 10.1007/s11606-024-08852-1 (PMC11436614; doi:10.1007/s11606-024-08852-1)
Supplement: Supplementary file 1 — Supplementary materials (DOCX 1744 KB) [file 11606_2024_8852_MOESM1_ESM.docx]

**Racial and Ethnic Disparities in Occult Hypoxemia Prevalence and Clinical Outcomes Among Hospitalized Patients: A Systematic Review and Meta-Analysis**

*Supplemental Materials*

[**Appendix A: Eligibility Criteria (PICOs)** 2](#_Toc163468428)

[**Appendix B: Search Strategies** 3](#_Toc163468429)

[**Appendix C: Studies Excluded During Full-Text Screening** 5](#_Toc163468430)

[**Appendix D: Methods for Synthesizing Pulse Oximeter Accuracy Data** 6](#_Toc163468431)

[**Appendix E: Risk of Bias Assessments** 8](#_Toc163468432)

[**Appendix F: Forest Plots of Individual Study Estimates** 16](#_Toc163468433)

Preregistered protocol available on the PROSPERO registry: [CRD42023402152](https://www.crd.york.ac.uk/PROSPERO/display_record.php?RecordID=402152)

# **Appendix A: Eligibility Criteria (PICOs)**

| **Population** | Adult inpatients or outpatients of different races/ethnicities (self-reported) or skin pigmentation (measured using chromaticity/phototype scales, spectroscopy, or other objective assessment) |
| --- | --- |
| **Intervention** | Concurrent (within 10 minutes) measurement of oxygen saturation in arterial blood gas and by pulse oximetry |
| **Comparator** | Not applicable |
| **Outcomes** | Frequency/risk of occult hypoxemia (defined as arterial oxygen saturation ≤ 88% despite a pulse oximeter reading > 88%, or stricter criteria), *or*  Clinical outcomes (e.g., treatment eligibility recognition/delay, treatment dosing/timing, treatment discontinuation/discharge, in hospital mortality) |

# **Appendix B: Search Strategies**

| **Database** |  | **Search Statement** | |
| --- | --- | --- | --- |
| **MEDLINE**  **Origination to 1/29/2024** |  | ((over?estimat* or under?estimat* or occult or hidden or missed or bias* or discrepanc* or disparit* or differ* or detect* or classif* or identif* or occurence* or risk) adj4 (hypoxia or hypoxemia or oximet*)).ti,ab. | 9171 |
|  |  | exp Ethnicity/ or exp Minority Groups/ or exp Skin Pigmentation/ or (((cutaneous or skin) adj3 (color* or colour* or pigment* or tone* or type)) or race or racial or ethnic* or ((minorit* or biracial or multiracial or african* or afro-american* or asian* or asiatic or black* or caucasian* or hispanic* or indian* or indigenous or latin* or native* or nonwhite* or (pacific adj3 islander*) or white) adj2 (patient* or participant* or adult*)) or ((persons or people) adj3 (color or colour))).ti,ab. | 455862 |
|  |  | 1 and 2 | 137 |
|  |  | exp Pediatrics/ or (pediatric* or paediatric* or neonat* or infant* or child* or adolescen*).ti,ab. | 2563586 |
|  |  | 3 not 4 | 113 |
|  |  | **Database total before deduplication** | **113** |
| **Embase**  **Origination to 2/5/2024** |  | ((over$estimat* or under$estimat* or occult or hidden or missed or bias* or discrepanc* or disparit* or differ* or detect* or classif* or identif* or occurence* or risk) NEAR/4 (hypoxia or hypoxemia or oximet*)):ti,ab | 12816 |
|  |  | 'ethnicity'/exp | 124747 |
|  |  | 'ethnic group'/exp | 227212 |
|  |  | 'minority group'/exp | 69859 |
|  |  | 'skin color'/exp | 25628 |
|  |  | (cutaneous or skin) NEAR/3 (color* or colour* or pigment* or tone* or type) | 49916 |
|  |  | race or racial or ethnic* | 595396 |
|  |  | ((minorit* or biracial or multiracial or african* or 'afro american*' or asian* or asiatic or black* or caucasian* or hispanic* or indian* or indigenous or latin* or native* or nonwhite* or white) NEAR/2 (patient* or participant* or adult*)):ti,ab | 135903 |
|  |  | pacific NEAR/3 islander* | 11294 |
|  |  | patient*:ti,ab or participant*:ti,ab or adult*:ti,ab | 14368879 |
|  |  | #9 and #10 | 6994 |
|  |  | ((persons or people) NEAR/3 (color or colour)):ti,ab | 2339 |
|  |  | #2 or #3 or #4 or #5 or #6 or #7 or #8 or #11 or #12 | 907551 |
|  |  | #1 and #13 | 254 |
|  |  | 'pediatrics'/exp | 140396 |
|  |  | pediatric*:ti,ab or paediatric*:ti,ab or neonat*:ti,ab or infant*:ti,ab or child*:ti,ab or adolescen*:ti,ab | 3382516 |
|  |  | #15 or #16 | 3405347 |
|  |  | #14 not #17 | 210 |
|  |  | **Database total before deduplication** | **210** |
| **CINAHL**  **Origination to 1/29/2024** |  | TI ( ((over?estimat* or under?estimat* or occult or hidden or missed or bias* or discrepanc* or disparit* or differ* or detect* or classif* or identif* or occurence* or risk) N4 (hypoxia or hypoxemia or oximet*)) ) OR AB ( ((over?estimat* or under?estimat* or occult or hidden or missed or bias* or discrepanc* or disparit* or differ* or detect* or classif* or identif* or occurence* or risk) N4 (hypoxia or hypoxemia or oximet*)) ) | 1746 |
|  |  | ( (MH "Ethnic Groups") or (MH "Minority Groups+") or (MH "Skin Pigmentation") ) OR TI ( (((cutaneous or skin) N3 (color* or colour* or pigment* or tone* or type)) or race or racial or ethnic* or ((minorit* or biracial or multiracial or african* or afro-american* or asian* or asiatic or black* or caucasian* or hispanic* or indian* or indigenous or latin* or native* or nonwhite* or (pacific N3 islander*) or white) N2 (patient* or participant* or adult*)) or ((persons or people) N3 (color or colour))) ) OR AB ( (((cutaneous or skin) N3 (color* or colour* or pigment* or tone* or type)) or race or racial or ethnic* or ((minorit* or biracial or multiracial or african* or afro-american* or asian* or asiatic or black* or caucasian* or hispanic* or indian* or indigenous or latin* or native* or nonwhite* or (pacific N3 islander*) or white) N2 (patient* or participant* or adult*)) or ((persons or people) N3 (color or colour))) ) | 181169 |
|  |  | 1 and 2 | 71 |
|  |  | (MH "Pediatrics+") OR TI ( (pediatric* or paediatric* or neonat* or infant* or child* or adolescen*) ) OR AB ( (pediatric* or paediatric* or neonat* or infant* or child* or adolescen*) ) | 941484 |
|  |  | 3 not 4 | 57 |
|  |  | **Database total before deduplication** | **57** |
| ***Total*** | | | **380** |
| ***Total after deduplication*** | | | **243** |

# **Appendix C: Studies Excluded During Full-Text Screening**

| **Citation** | **Exclude Reason** |
| --- | --- |
| Gadrey SM, Mohanty P, Haughey SP, Jacobsen BA, Dubester KJ, Webb KM, et al. Overt and occult hypoxemia in patients hospitalized with COVID-19. *Critical Care Explorations*. 2023;5(1):e0825. | Ineligible outcome |
| Gottlieb ER, Ziegler J, Morley K, Rush B, Celi LA. Assessment of racial and ethnic differences in oxygen supplementation among patients in the intensive care unit. *JAMA Internal Medicine.* 2022;182(8): 849-858 | Ineligible outcome |
| Gudelunas MK, Lipnick M, Hendrickson C, Vanderburg S, Okunlola B, Auchus, I, et al. Low perfusion and missed diagnosis of hypoxemia by pulse oximetry in darkly pigmented skin: a prospective study. *Anesthesia and Analgesia.* 2023;138(3): 552-561. | Ineligible intervention/exposure |
| Rovinski R, Grealis K, Kumar N, Velasquez-Duran M, Patel S, Ferreira TB, Gershengorn HB, Mallow C. Hemodynamic variations are associated with increased odds of discrepancy between pulse oximeter and arterial oxygen saturation. *American Journal of Respiratory and Critical Care Medicine*. 2023;207(1). | Ineligible publication type |
| Stannard B, Burnett GW, Lin, HM, Ouyang Y, Levin MA. Intraoperative occult hypoxemia is an independent risk factor for 30-day and 1-year mortality. *Anesthesia and Analgesia*. 2023;136(4): 33. | Ineligible publication type |

# **Appendix D: Methods for Synthesizing Pulse Oximeter Accuracy Data**

Pulse oximeter accuracy data were available from 11 included studies (1–11). Reported precision estimates for each race or ethnicity group in each study were adjusted using the formula below (12) when the number of patients in each group was reported. The purpose of this adjustment, which was also implemented by Shi et al (13), was to produce more conservative estimates of precision that account for repeated observations of the same patient. The number of paired oximetry measurements per patient was calculated by dividing the total number of oximetry measurements by the number of patients (e.g., in a group of 50 patients in which 500 paired measurements were collected, the approximate number of measurements per patient would be 500/50 = 10 measurements).

$$Adjusted SD=\sqrt{{Reported SD}^{2} \times\left( \frac{Total Measurements-1}{Total Measurements-Measurements per Patient} \right)}$$

To synthesize mean bias and precision data, we employed the same general approach used in occult hypoxemia prevalence analyses and in the meta-analyses reported by Shi et al (13) to account for dependencies among estimates. This consisted of using hierarchical random-effects models, cluster-robust confidence intervals, and degrees of freedom calculated using the Satterwaithe approximation. Further detail on these methods is provided in the main article.

References

1. Bangash MN, Hodson J, Evison F, Patel JM, Johnston AM, Gallier S, et al. Impact of ethnicity on the accuracy of measurements of oxygen saturations: a retrospective observational cohort study. *EClinicalMedicine*. 2022;48. https://doi.org/10.1016/j.eclinm.2022.101428.

2. Burnett GW, Stannard B, Wax DB, Lin HM, Pyram-Vincent C, DeMaria S, et al. Self-reported race/ethnicity and intraoperative occult hypoxemia: a retrospective cohort study. *Anesthesiology*. 2022;136(5): 688–696. https://doi.org/10.1097/ALN.0000000000004153.

3. Chesley CF, Lane-Fall MB, Panchanadam V, Harhay MO, Wani AA, Mikkelsen ME, et al. Racial disparities in occult hypoxemia and clinically based mitigation strategies to apply in advance of technological advancements. *Respiratory Care*. 2022;67(12): 1499–1507. https://doi.org/10.4187/respcare.09769.

4. Garnet B, Diaz-Lankenau R, Jean E, Campos M. Accuracy of pulse oximetry for long-term oxygen therapy assessment in chronic obstructive pulmonary disease. *Annals of the American Thoracic Society*. 2023;20(11): 1587–1594. https://doi.org/10.1513/AnnalsATS.202209-837OC.

5. Kalra A, Shou BL, Zhao D, Wilcox C, Keller SP, Whitman GJR, et al. Racial and ethnical discrepancy in hypoxemia detection in patients on extracorporeal membrane oxygenation. *JTCVS Open*. 2023;14: 145–170. https://doi.org/10.1016/j.xjon.2023.02.011.

6. Kalra A, Wilcox C, Holmes SD, Tonna JE, Jeong IS, Rycus P, et al. *Characterizing the racial discrepancy in hypoxemia detection in VV-ECMO: an ELSO registry analysis*. 2023. https://doi.org/10.21203/rs.3.rs-3617237/v1.

7. Seitz KP, Wang L, Casey JD, Markus SA, Jackson KE, Qian ET, et al. Pulse oximetry and race in critically ill adults. *Critical Care Explorations*. 2022;4(9): e0758. https://doi.org/10.1097/CCE.0000000000000758.

8. Sudat SEK, Wesson P, Rhoads KF, Brown S, Aboelata N, Pressman AR, et al. Racial disparities in pulse oximeter device inaccuracy and estimated clinical impact on COVID-19 treatment course. *American Journal of Epidemiology*. 2023;192(5): 703–713. https://doi.org/10.1093/aje/kwac164.

9. Valbuena VSM, Seelye S, Sjoding MW, Valley TS, Dickson RP, Gay SE, et al. Racial bias and reproducibility in pulse oximetry among medical and surgical inpatients in general care in the Veterans Health Administration 2013-19: multicenter, retrospective cohort study. *BMJ*. 2022;378: e069775. https://doi.org/10.1136/bmj-2021-069775.

10. Valbuena VSM, Barbaro RP, Claar D, Valley TS, Dickson RP, Gay SE, et al. Racial bias in pulse oximetry measurement among patients about to undergo extracorporeal membrane oxygenation in 2019-2020: a retrospective cohort study. *Chest*. 2022;161(4): 971–978. https://doi.org/10.1016/j.chest.2021.09.025.

11. Wong AKI, Charpignon M, Kim H, Josef C, de Hond AAH, Fojas JJ, et al. Analysis of discrepancies between pulse oximetry and arterial oxygen saturation measurements by race and ethnicity and association with organ dysfunction and mortality. *JAMA Network Open*. 2021;4(11): e2131674. https://doi.org/10.1001/jamanetworkopen.2021.31674.

12. Tipton E, Shuster J. A framework for the meta-analysis of Bland–Altman studies based on a limits of agreement approach. *Statistics in Medicine*. 2017;36(23): 3621–3635. https://doi.org/10.1002/sim.7352.

13. Shi C, Goodall M, Dumville J, Hill J, Norman G, Hamer O, et al. The accuracy of pulse oximetry in measuring oxygen saturation by levels of skin pigmentation: a systematic review and meta-analysis. *BMC Medicine*. 2022;20(1): 267. https://doi.org/10.1186/s12916-022-02452-8.

# **Appendix E: Risk of Bias Assessments**

| **Study Name or Author Year** | **Study Participation** | **Study Attrition** | **Prognostic Factor Measurement** | **Outcome Measurement** | **Study Confounding** | **Statistical Analysis and Reporting** | **Overall Risk of Bias** |
| --- | --- | --- | --- | --- | --- | --- | --- |
| **Bangash 2022** | Low  Included all patients for whom paired measurements and ethnicity were recorded. | Low  Retrospective cohort study. Included all consecutive measurement during the specified time period. | High  Used self-reported race or ethnicity as a proxy for pigmentation. | Moderate  Occult hypoxemia defined as SaO2 less than 94%% despite SpO2 greater than 94%. Different methods used for recording SaO2 and SpO2 (automatically updated in EHR vs manual entry by health care professional). | Moderate  GAM model controlled for several clinical and demographic factors that were potential confounders. Do not account for all potential confounding variables (*eg*, socioeconomic factors). | Low  GAM model controlled for potential confounders. | **Moderate** |
| **Burnett 2022** | Low  Included all patients who received an anesthetic with at least 1 ABG sample during the time period of the study. | Low  Retrospective cohort study. Appear to include all data where both measurements were identified during the specified time period. | High  Used self-reported race or ethnicity obtained from medical record data as a proxy for pigmentation. | Low  Occult hypoxemia defined as SaO2 less than 88% despite SpO2 greater than 92%. SaO2 measurement was the same for all patients and changes in SpO2 devices were accounted for by year measurement was taken. | Moderate  Used a multivariable model which controlled for all of the collected demographic, comorbidity, and operative variables. Do not account for all potential confounding variables (*eg*, socioeconomic factors). | Low  GEE modeling was used to determine if race was an independent predictor of occult hypoxemia. No evidence of selective reporting of results. | **Moderate** |
| **Chelsey 2022** | Low  Included all critically ill patients with paired measurements within 10 minutes of each other during the study period. | Low  Retrospective cohort study. Some analyses only included Black and White patient groups due to sample size, otherwise all patients appear to be included in analyses. | High  Used self-reported race or ethnicity (White, Other, Hispanic/Latinx, Black, Asian) as a proxy for pigmentation. | Low  Occult hypoxemia defined as SaO2 < 88% when pulse oximeter oxygen saturation was between 92–96%. Sites used different blood gas analyzers, and oximeters but there were no differences between sites when compared. | Moderate  Multivariable model controlled for a limited number of potential confounders (age, sex, hemoglobin). | Low  Used multivariable logistic regression model to examine the association between self-reported race and occult hypoxemia. No evidence of selective reporting of results. | **Moderate** |
| **Fawzy 2022** | Low  Included patients for whom specified data were available during the study period. | Low  Retrospective cohort study. Flow diagrams shows reasons for exclusion of patients from each analysis and exclusions are appropriate. | High  Used self-reported race or ethnicity (Asian, Black/African American, White and Hispanic or non-Hispanic) as a proxy for pigmentation. | Moderate  All ABG samples were analyzed via CO-oximetry using an ABL brand device. PO device type and reading location was not reported and likely varied between sites. Delayed treatment recognition was defined as those patients with a predicted SaO2 of 94% or less before a measured SpO2 of 94% or less or oxygen treatment initiation. Unrecognized treatment eligibility was defined as those patients with a predicted SaO2 of 94% or less who did not initiate treatment with oxygen or have a recorded SpO2 of 94% or less at any time. | Moderate  Model was adjusted for covariates that captured disease severity or an underlying comorbidity or had a known or theoretical association with PO accuracy, including demographic characteristics along with time-varying clinical and laboratory variables. Do not account for all potential confounding variables (*eg*, socioeconomic factors). | Moderate  The difference in time to recognition of treatment eligibility between patients of racial and ethnic minority groups and White patients was estimated using a Cox proportional hazards model. Among individuals with delayed recognition of treatment eligibility, Wilcoxon rank sum tests were used to compare the distributions of length of delayed recognition between groups. Only patients with complete data on covariates were included in adjusted model. No evidence of selective reporting of results. | **Moderate** |
| **Fawzy 2023** | Low  Retrospective study that included data from all patients from an electronic health record database of COVID-19 patients who had at least 1 SaO_2_ measurement recorded. | Low  Retrospective cohort study. Appear to include all patients in analyses. | High  Investigated the effect of self-reported race or ethnicity rather than skin pigmentation. | Moderate  Occult hypoxemia defined as SaO_2_ levels below 88% with concurrent SpO_2_ between 92% and 96%. For each SaO_2_ value, the nearest SpO_2_ value within 10 minutes was used. Information on devices not provided; likely varied between sites. Unrecognized need for treatment defined as having an initial SaO_2_ measurement below 94% despite a 10-minute SpO_2_ of 94%or higher. | Moderate  Adjusted for several potential confounders, including age, sex, maximum WHO criteria, BMI at admission, current smoking status, and CCI. Other potential confounding variables not accounted for. | Low  The association of race and ethnicity with error in pulse oximetry was examined using a linear mixed effects model. Clustering due to repeated measures within-patient was accounted for using a random intercept and hospital site. Only the first hospital stay and records without missing covariate data were included in the adjusted model. The association between race and ethnicity and unrecognized need  for COVID-19 therapy and associations of unrecognized need for treatment with in-hospital mortality and hospital readmissions were examined with mixed effects logistic regression models. The association with time to receipt of COVID-19 therapy was examined using a mixed effects Cox proportional hazard model. No evidence of selective reporting of results. | **Moderate** |
| **Garnet 2023** | Low  Retrospective study that included patients who underwent home oxygen evaluation between January 2012 and December 2019 with COPD and concurrent SaO_2_ and SpO_2_ measurements. Excluded evaluations that occurred within 30 days of changes in corticosteroid medication or discharge from a hospitalization related to a different lung condition. | Low  Retrospective cohort study. Appear to include all patients in analyses. | High  Investigated the effect of self-reported race and ethnicity rather than skin pigmentation. | Low  At-home evaluation entailed measuring SpO_2_ on room  air using a finger sensor and a Life Sense monitor after  waiting 30 seconds, as well as confirmation of adequate waveform with simultaneous  ABG analysis with co-oximetry. Same devices used across patients. | Moderate  Multivariate linear regression model included age, race, smoking status, heart failure, PaO2, and arterial carbon dioxide pressure as covariates. Other potential confounding variables not accounted for. | Low  Percentage of false negatives and occult hypoxemia were reported separately for Black patients. Bias was examined with a multivariate linear regression model with race as an independent variable. No evidence of selective reporting of results. | **Moderate** |
| **Henry 2022** | Low  Retrospective study that included adults meeting criteria from 4 self-identified racial groups with paired measurements. | Low  Appear to include all patients in analysis. | High  Patients were categorized into 4 self-identified racial groups. | Moderate  PO readings and blood samples were done simultaneously (*ie*, zero minutes of separation). Method of blood gas analysis and pulse oximetry not reported and may have differed between study sites. Occult hypoxemia was defined as SaO2 less than 88% despite a normal SpO2 (*ie*, ≥ 92%). Hospital-free days were counted as the number of days alive and out of hospital following the index time through 28 days of follow-up. | Moderate  Adjusted for select potential confounders in both analysis of occult hypoxemia (age, mean arterial pressure less than 65 mmHg or the use of continuous infusions of IV vasopressors at the time of SaO2 assessment, and presence of COPD or home oxygen use) and analysis of treatment outcomes (age, sex, COPD or home oxygen use, index location [ICU vs surgical], and acuity of illness). | Low  GEE was used to account for multiple observations. No evidence of selective reporting. | **Moderate** |
| **Kalra 2023** | Low  Retrospective study of patients undergoing ECMO at a tertiary care center between June 2016 and April 2021. Patients without race or ethnicity information or SpO2 and SaO2 data were excluded. | Low  Datapoints were excluded if oxygen saturation ≤ 70% or SpO_2_–SaO_2_ pairs were not measured within 10 minutes. | High  Investigated the effect of self-reported race and ethnicity rather than skin pigmentation. | Moderate  Occult hypoxemia  was defined as SaO_2_ < 88% with a time matched SpO_2_ ≥ 92%. ABGs were collected every 2 to 4 hours during ECMO support, and SpO2 was recorded every 15 minutes, according to the standard clinical protocol. SpO2 and SaO2 measurements were recorded as a single reading at a particular time and  date, measured ≤ 10 minutes apart. Depending on ECMO type, the pulse oximeter probe was placed on either the right finger or right earlobe or right or left hand. Device info not provided but data was obtained from a single site. | Moderate  Adjusted model controlled for demographics and time-dependent laboratory and clinical variables (pH, temperature, lactate dehydrogenase, and hemoglobin). Covariates were age, sex, vasopressor or inotrope requirement during ECMO, and cannulation strategy. | Low  Bland–Altman analyses  and linear mixed-effects modeling, adjusting for prespecified covariates, were used to assess the SpO2–SaO2 discrepancy between races/ethnicities. No evidence of selective reporting. | **Moderate** |
| **Kalra 2023 (Characterizing)** | Low  Retrospective study of adults with venovenous ECMO who had data on race or ethnicity from a large, multisite database between January 2018 and May 2023. | Low  Excluded repeat ECMO runs within individual patients, patients without data on hypoxemia at either time point, and patients with extreme outlier values for the  difference between SpO2 and SaO2. | High  Investigated the effect of self-reported race or ethnicity rather than skin pigmentation. | Moderate  The pre-ECMO ABG that was closest to the start of ECMO cannulation was chosen, and the on-ECMO ABG closest to 24 hours after the start of cannulation was selected. Occult hypoxemia was defined as SaO2 ≤ 88% with a time matched SpO2 ≥ 92%. No detail provided on SpO2 measurement, which likely differed across sites. Timing of SpO2 measurement relative to ABG measurement is unclear. | Moderate  Covariates in both the pre-ECMO and on-ECMO models  included age, sex, presence of pre-ECMO temporary mechanical circulatory support, and presence of pre-ECMO vasopressor and inotrope infusions. The on-ECMO model also included hemolysis,  hyperbilirubinemia, cannulation strategy, ECMO pump flow rate, and on-ECMO serum lactate value. Other potential confounders were not accounted for. | Low  SpO2-SaO2 differences were compared with Kruskal-Wallis and  Wilcoxon rank-sum tests. Bland-Altman analyses were used to assess agreement between SpO2 and SaO2. Multivariable logistic regressions were performed to examine whether race or ethnicity was associated with occult hypoxemia in pre-ECMO and on-ECMO measurements. No evidence of selective reporting. | **Moderate** |
| **Seitz 2022** | Low  Retrospective study that included all patients meeting criteria with paired measurements and race documented as Black or White during the study period. Excluded patients with COVID-19. Patients with other values for race were not included due to an inadequate number of patients for comparison. | Low  Retrospective study and all patients included in study were included in analysis. | High  Race (Black or White) was used as a proxy for skin pigmentation | Low  Measurements occurred within 10 minutes of each other and the SpO2 value closest in time was used. SaO2 and SpO2 measurements were done using the same devices for all patients. Occult hypoxemia was defined as SaO2 < 88% with SpO2 values of 92–96%. | Moderate  Did not appear to examine occult hypoxemia with a multivariate model, but race groups comparable on demographic and clinical variables with the exception of ECMO; sensitivity analysis conducted excluding observations from patients on ECMO. | Low  Compared rate of occult hypoxemia between groups. No evidence of selective reporting. | **Moderate** |
| **Sjoding 2020** | Moderate  Retrospective study; does not clearly describe how patients were selected for inclusion. | Low  Retrospective study. | High  Self-reported race was used as a proxy for skin pigmentation | Moderate  Unknown whether all SpO2 measurements were taken with different oximeters. Paired measurements occurred within 10 minutes of each other. Analyses limited to measures of arterial blood gas that included carboxyhemoglobin and methemoglobin saturations to ensure that arterial oxygen saturation was directly measured by co-oximetry. | Moderate  Proportion of patients with occult hypoxemia adjusted for age, sex, and cardiovascular score on the Sequential Organ Failure Assessment (SOFA), but only in one of two cohorts. | Low  Cluster-robust confidence intervals used to account for repeated paired measurements within patients. | **Moderate** |
| **Sudat 2022** | Low  Appear to include all patients meeting their criteria during the study period. Excluded patients who were not non-Hispanic Black or non-Hispanic White. Second cohort included ED patients with COVID-19. Excluded visits with no documented SpO2. | Low  Retrospective study; appear to include all patients meeting their criteria in the respective cohort analyses. | High  Race (Black or White) was used as a proxy for skin pigmentation. | Moderate  Paired each SaO2 measurement with the nearest recorded SpO2 for the same person, truncated at ± 10m from the earlier of the ABG specimen time or result time. No information provided on ABG or PO measurement. Treatment outcomes (time spent in ED, hospital admission, dexamethasone administration and timing, oxygen supplementation and timing, return to the hospital after discharge home) from EHR data. Defined hypoxemia as an SaO2 < 90%. | Moderate  Important differences were noted between NHB and NHW groups at baseline (homelessness, insurance types, comorbidities), but these and additional demographic and clinical covariates were included in the model. Do not account for all possible confounders. | Low  Used G-computation to build 2 counterfac­tuals to assess the possible impacts of differential SpO2 measurement error on COVID-19-related outcomes for NHB patients. Computed the mean difference between the pre­dicted outcome with the observed SpO2 values and the pre­dicted outcome with SpO2 values shifted by the measurement difference from the initial PO bias analysis. Also compared prevalence of OH between groups and reported p value. No evidence of selective reporting. | **Moderate** |
| **Valbuena 2022 ECMO** | Moderate  Retrospective study in­cluding adult patients with ARDS or COVID-19 on ECMO for respir­atory failure during the study period. Included patients with relevant data (blood gas samples had to meet certain criteria for timing). For hypoxemia analyses, only included race or ethnicity categories that met their calculated sample size threshold (N ≥ 400). Excluded a large number of patients for this reason, including groups where patients may have had more pigmented skin (*eg*, North African). | Low  Retrospective study using registry data. | High  Race or ethnicity was used as a proxy for skin pigmentation. | Moderate  Measurements were “matched” but specific time interval not provided. No information provided on SaO2 or SpO2 measurement. Occult hypoxemia was defined as low arterial oxygen saturation (SaO2 ≤ 88%) on arterial blood gas measurement despite a pulse oximetry reading in the range of 92% to 96%. | Moderate  Multivariable model controlled for a limited number of potential confounders (sex and measured SpO2). | Low  Multivariable analyses were performed by logistic regression for each race and ethnicity group compared with White patients to examine the relationship between these variables with the odds of occult hypoxemia. No evidence of selective reporting. | **Moderate** |
| **Valbuena 2022 VHA** | Low  Retrospective study including all SpO2 and SaO2 data available for hospital stays, with some exclusions for indicators of critical illness (to capture a general hospital sample, not ICU). Valid records in the database require core identifiers, including race and ethnic origin, to be present. | Low  Retrospective study and all patients included in study were included in analysis, although Hispanic patients were not included in all analyses. | High  Race (Black, Hispanic, or White) was used as a proxy for skin pigmentation. | Moderate  Included pairs of measurements occurring with 10 minutes of one another. No information on device/location for PO and SaO2 measurements was available. Occult hypoxemia was defined as defined as arterial SaO2 <88% despite a SpO2 reading of ≥92%. | Moderate  Models were adjusted for patient level characteristics that included age, sex, patient comorbidities, supplemental oxygen, and diagnoses on admission. Do not account for all potential confounders. | Low  Fit a multivariable logistic regression model to predict the odds of occult hypoxemia. No evidence of selective reporting. | **Moderate** |
| **Wong 2021** | Low  Retrospective study that included patients from all units available in the data sets with SpO2 measurements within the specified range and with self-identified race or ethnicity classified as Asian, Black, Hispanic, or White. | Low  Retrospective study. All included patients included in analysis; excluded patients from subgroup analysis when data on corresponding characteristics were missing. | High  Classified patients by race or ethnicity, not skin pigmentation. | Moderate  Each ABG-measured SaO2 was matched with the closest SpO2 value recorded within the previous 5 minutes. Do not report how blood gas analysis was conducted or device/location of PO measurement. Occult hypoxemia was defined as SpO2 > 88% but SaO2 < 88%. Clinical outcomes (in-hospital mortality, length of stay, organ dysfunction [SOFA scores], laboratory values) extracted from HER. | Moderate  Adjusted only for age, sex, SOFA score. | Low  Multivariate logistic regression was used for assessing binary end points, multivariate ordinal regression for numerical end points, and multivariate linear models for continuous end points, using analysis of variance to test for the impact of hidden hypoxemia while adjusting for other covariates. Calculated relative risk of OH by race or ethnicity. No evidence of selective reporting. | **Moderate** |

ABG = arterial blood gas; BMI=body mass index; CCI=Charlson Comorbidity Index; COPD = chronic-obstructive pulmonary disease; ECMO=extracorporeal membrane oxygenation; ED = emergency department; EHR = electronic health record; GEE = generalized estimating equation; ICU = intensive care unit; ITU = intensive treatment unit; PO = pulse oximeter; WHO=World Health Organization.

# **Appendix F: Forest Plots of Individual Study Estimates**

Note: Dashed gray line around overall RE model estimates corresponds to 95% prediction interval.

*Prevalence of Occult Hypoxemia (Black patients)*

*
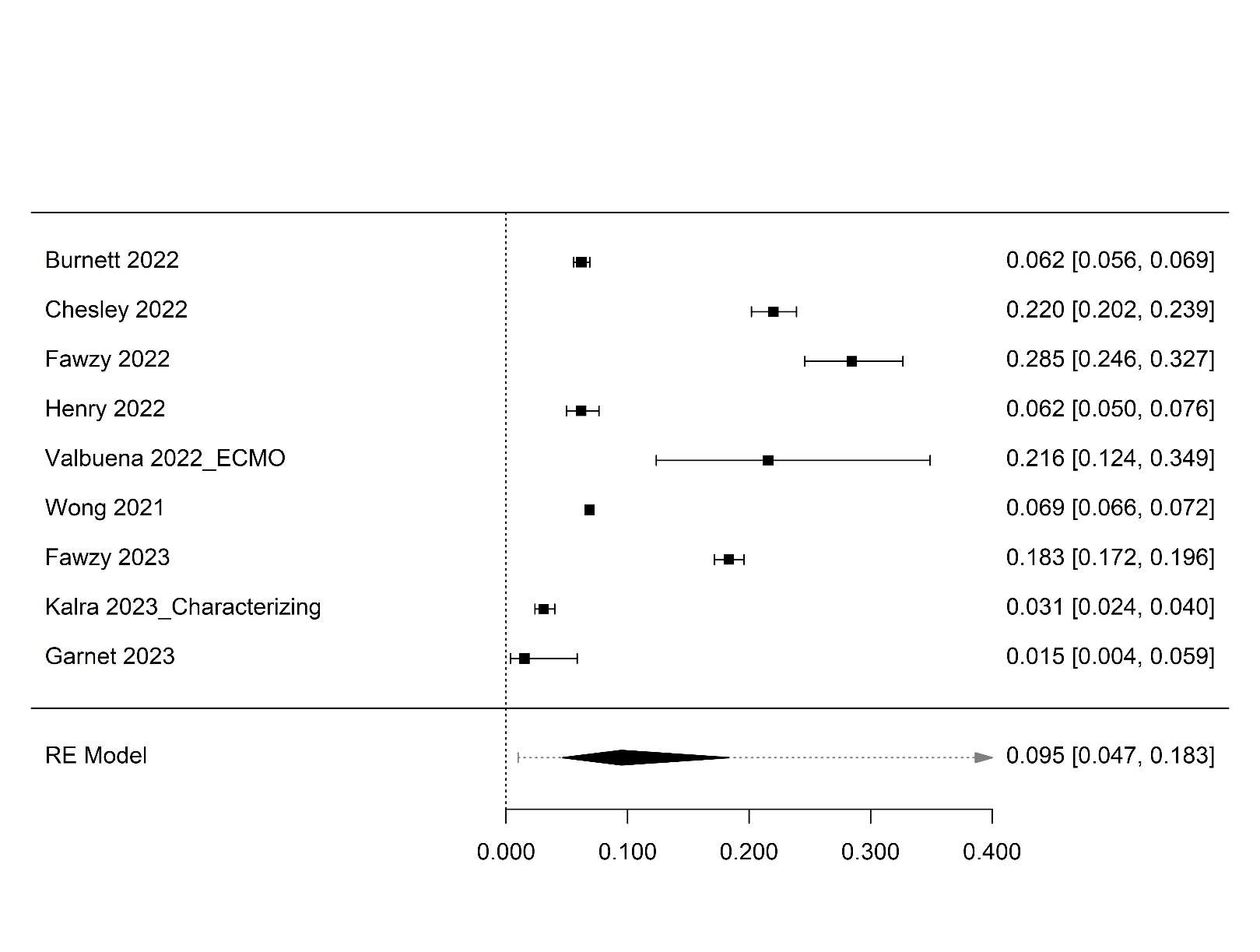
*

*Prevalence of Occult Hypoxemia (Black patients – Observation level)*

*
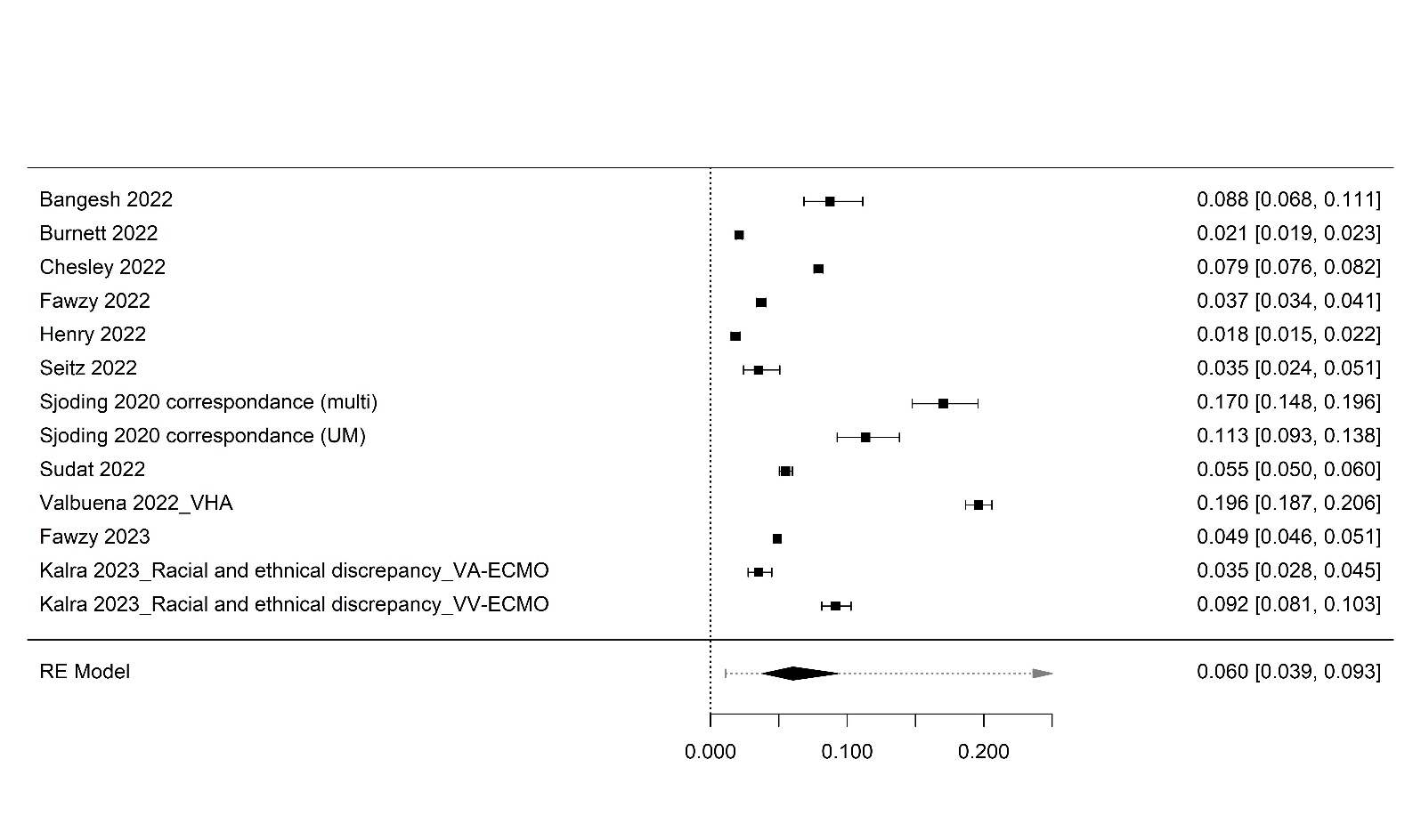
*

*Prevalence of Occult Hypoxemia (patients identifying as Asian, Latinx, Indigenous, multiracial, or other race or ethnicity)*

*
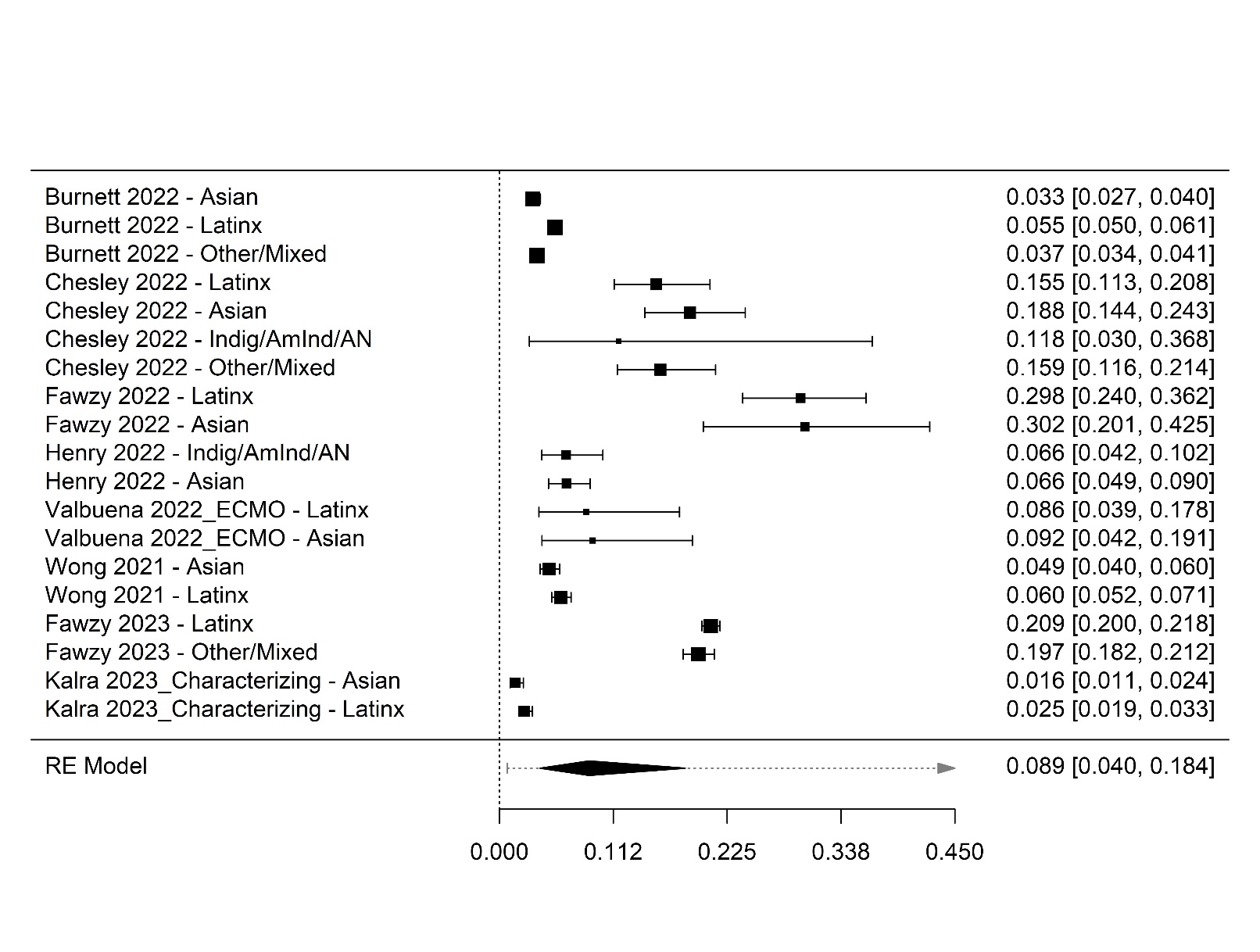
*

*Prevalence of Occult Hypoxemia (patients identifying as Asian, Latinx, Indigenous, multiracial, or other race or ethnicity – Observation Level)*

*
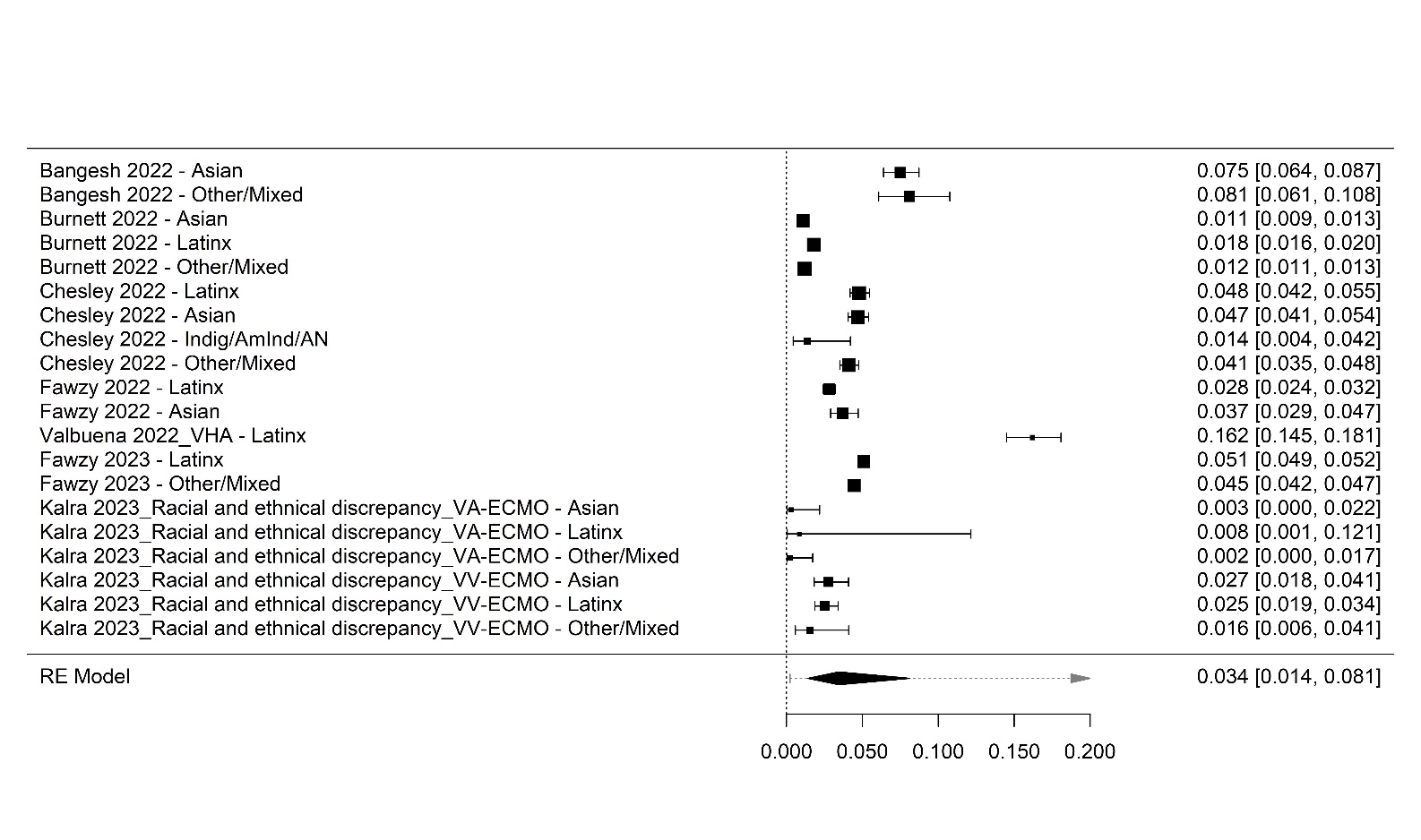
*

*Prevalence of Occult Hypoxemia (White patients)*

*
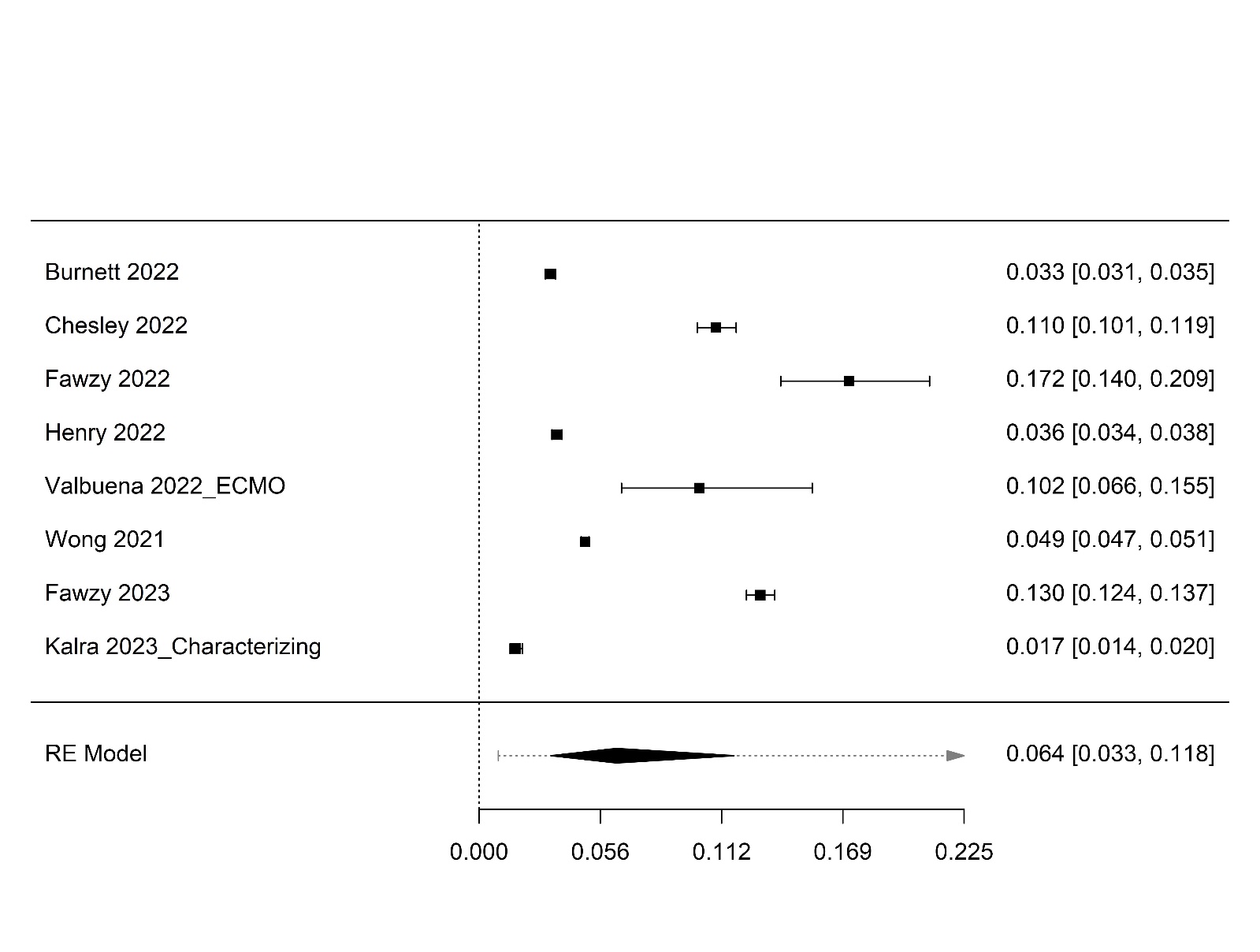
*

*Prevalence of Occult Hypoxemia (White patients – Observation level)*

*
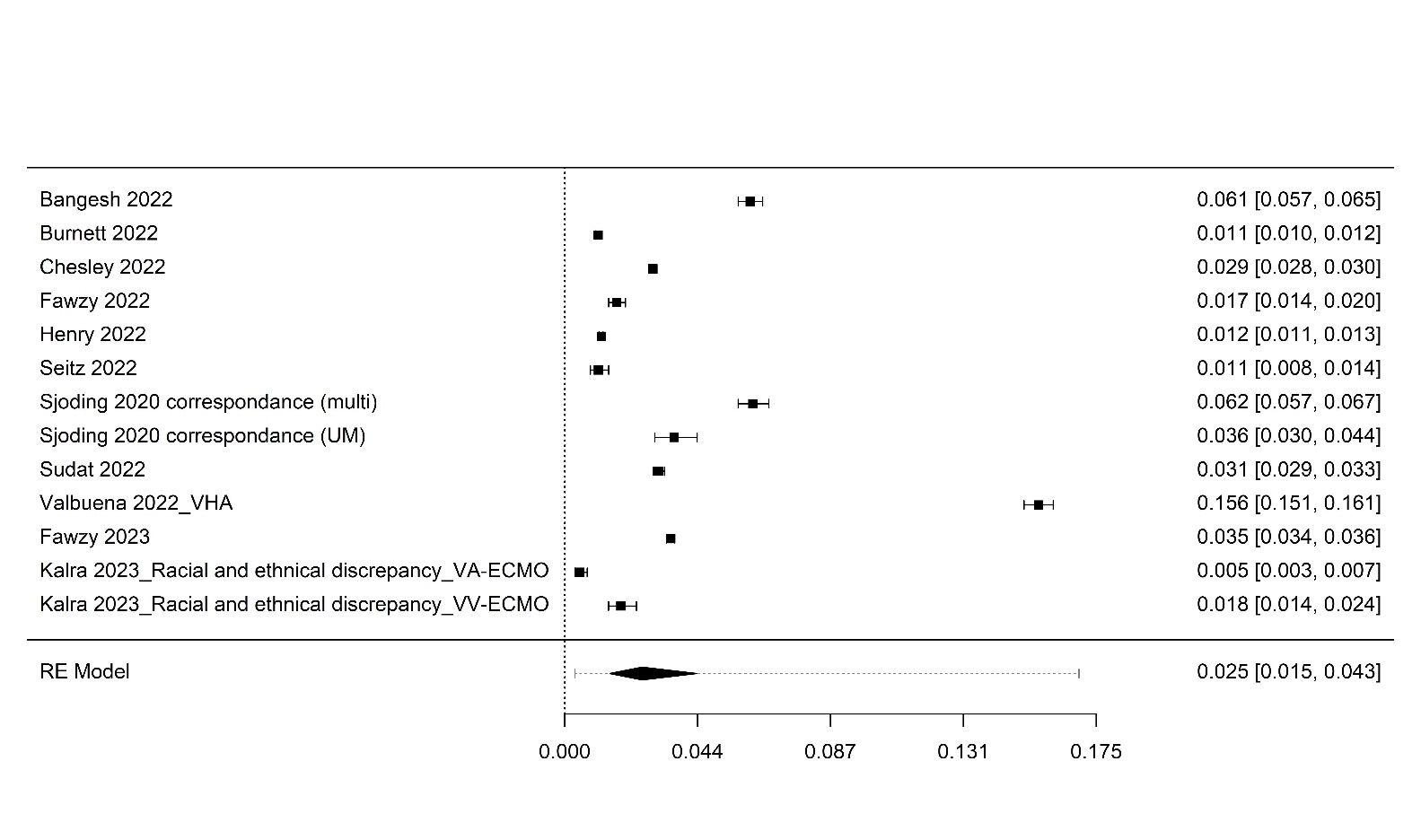
*

*Adjusted Odds of Occult Hypoxemia (Black patients; reference group is White patients)*

*
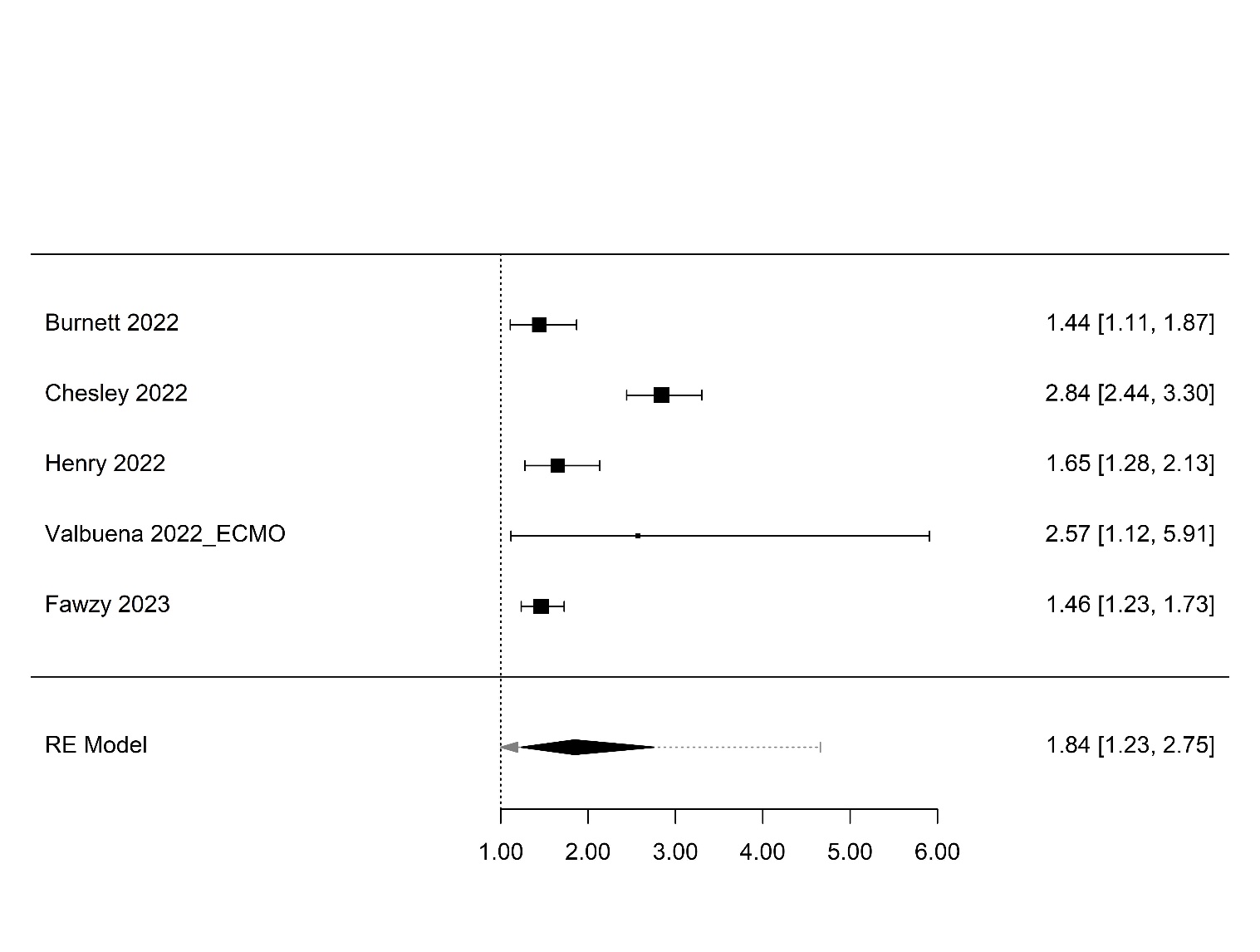
*

*Adjusted Odds of Occult Hypoxemia (patients identifying as Asian, Latinx, Indigenous, multiracial, or other race or ethnicity; reference group is White patients)*

*
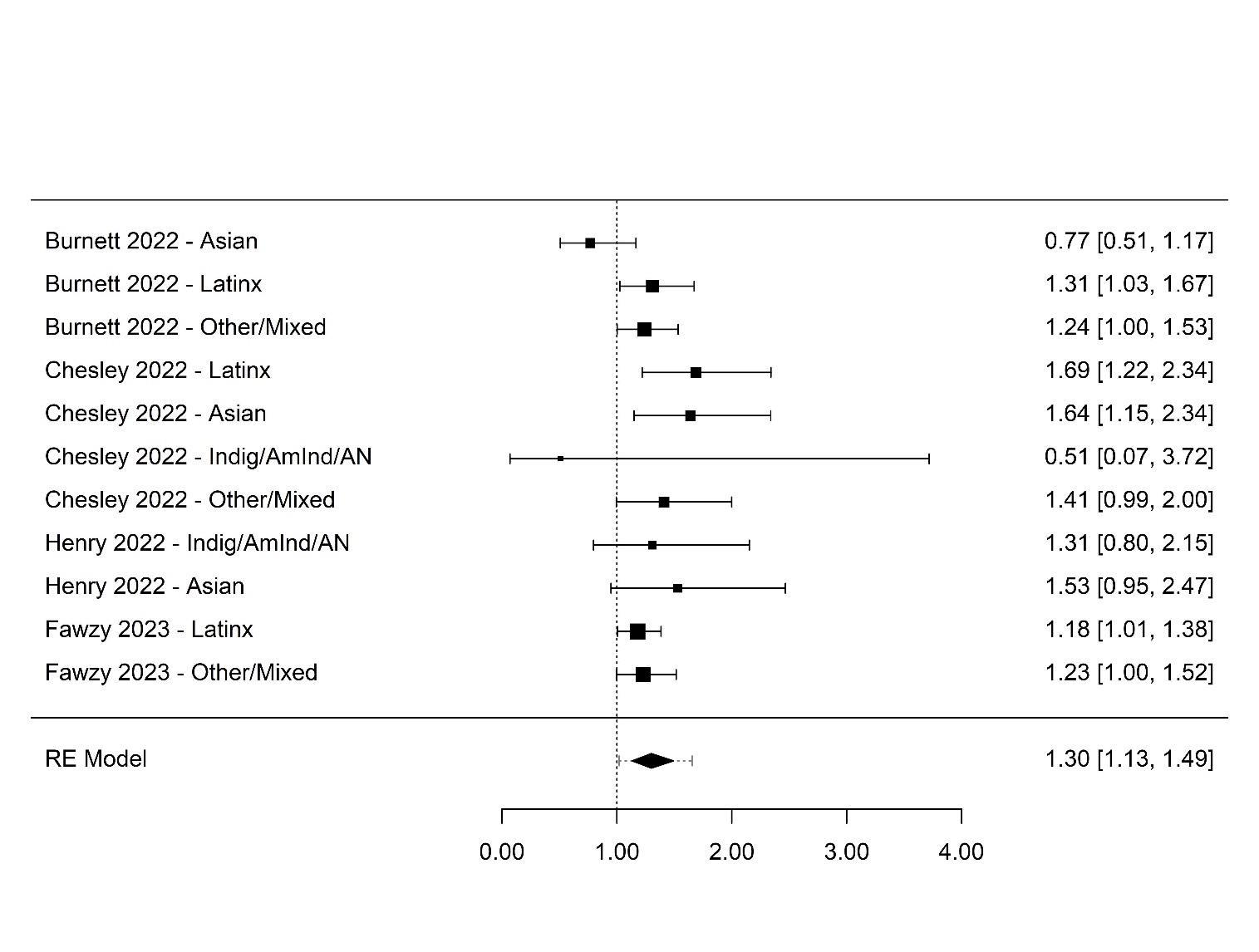
*
